# Unusual double ligand holes as catalytic active sites in LiNiO_2_

**DOI:** 10.1038/s41467-023-37775-4

**Published:** 2023-04-13

**Authors:** Haoliang Huang, Yu-Chung Chang, Yu-Cheng Huang, Lili Li, Alexander C. Komarek, Liu Hao Tjeng, Yuki Orikasa, Chih-Wen Pao, Ting-Shan Chan, Jin-Ming Chen, Shu-Chih Haw, Jing Zhou, Yifeng Wang, Hong-Ji Lin, Chien-Te Chen, Chung-Li Dong, Chang-Yang Kuo, Jian-Qiang Wang, Zhiwei Hu, Linjuan Zhang

**Affiliations:** 1 Key Laboratory of Interfacial Physics and Technology, Shanghai Institute of Applied Physics, Chinese Academy of Sciences, Shanghai, 201800 China; 2 National Synchrotron Radiation Research Center, Hsinchu, Taiwan, ROC; 3 Department of Physics, Tamkang University, New Taipei City, Taiwan, ROC; 4 Max Planck Institute for Chemical Physics of Solids, Dresden, 01187 Germany; 5 Department of Applied Chemistry, Ritsumeikan University, Kusatsu, Shiga, 535-8577 Japan; 6 Department of Electrophysics, National Yang Ming Chiao Tung University, Hsinchu, Taiwan, ROC; 7 University of Chinese Academy of Sciences, Beijing, 10049 China

**Keywords:** Electrocatalysis, Electrocatalysis, Catalytic mechanisms

## Abstract

Designing efficient catalyst for the oxygen evolution reaction (OER) is of importance for energy conversion devices. The anionic redox allows formation of O-O bonds and offers higher OER activity than the conventional metal sites. Here, we successfully prepare LiNiO_2_ with a dominant 3*d*^8^*L* configuration (*L* is a hole at O 2*p*) under high oxygen pressure, and achieve a double ligand holes 3*d*^8^*L*^2^ under OER since one electron removal occurs at O 2*p* orbitals for Ni^III^ oxides. LiNiO_2_ exhibits super-efficient OER activity among LiMO_2_, *R*MO_3_ (M = transition metal, *R* = rare earth) and other unary 3d catalysts. Multiple in situ/operando spectroscopies reveal Ni^III^→Ni^IV^ transition together with Li-removal during OER. Our theory indicates that Ni^IV^ (3*d*^8^*L*^2^) leads to direct O-O coupling between lattice oxygen and *O intermediates accelerating the OER activity. These findings highlight a new way to design the lattice oxygen redox with enough ligand holes created in OER process.

## Abstract (web summary)

Lattice-oxygen redox is pivotal for high oxygen evolution reaction (OER) activity. Here, LiNiO_2_, a unary 3*d*-transition metal oxide catalyst, exhibits superefficient activity during the OER due to the creation of double O 2*p* holes states, according to operando XAS, XRD, and Raman spectroscopy observations.

## Introduction

The electrocatalysis of the oxygen evolution reaction (OER) is at the core of many energy conversion devices, such as water and CO_2_ electrolysers, and metal-air batteries^1–3^. Because of the sluggish kinetics of OER, the exploration of OER electrocatalysts with high intrinsic activity plays a decisive role in determining the device performance, and thus the fundamental understanding of OER mechanisms is essential^4–7^. The conventional adsorbate evolution mechanism, involving multiple adsorbed intermediates with highly correlated adsorption strengths, exhibits a minimum theoretical overpotential of about 0.37 V^8,9^. This limitation has been circumvented by a recently identified mechanism^10,11^—the lattice oxygen oxidation mechanism—wherein the oxygen ligands are electrochemically activated, coupled directly with *O intermediates, and released from the lattice matrix^4^. Lattice-oxygen-activated electrocatalysts, such as 3*d* late-transition-metal (TM) oxides with high metal oxidation states, universally feature a high TM-O covalency with a strong orbital hybridization between TM 3*d* and O 2*p* orbitals, which enables intramolecular electron transfer from the oxygen ligands to the TM cations, leaving ligand holes for lattice oxygen activation^4,10^. Such an anionic redox mechanism is well documented in lithium-ion-battery (LIB) cathodes^12^ and thermal oxidative catalysis^13^.

**Fig. 1:**
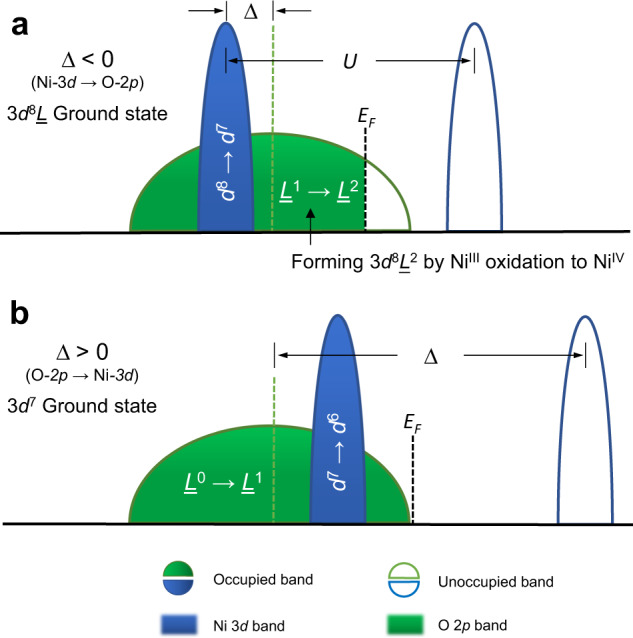
Schematic single-electron removal spectra of Ni^III^ oxides with a formal 3*d*^7^ filling. **a** *Δ* < 0 and **b** *Δ* > 0 scenarios. Charge-transfer energy *Δ*: energy cost for electron transferring from the O 2*p* band to the Ni 3*d* band, and *U*: energy cost for electron transferring from the occupied 3*d* band to the unoccupie*d* 3*d* band. The hybridization between Ni 3*d* and O 2*p* is neglecte*d* for sim*p*licity. The Fermi level (E_F_) is shown as a vertical dash black line and the O 2*p* band is used as the energy reference considering the position of E_F_ is arbitrary in the band gap.

Ni^III^ oxides are intrinsically anionic-redox-active. Here we use the notation Ni^III^ rather than Ni^3+^ as a reminder that the formally trivalent Ni ion is in a highly covalent situation where holes in the oxygen 2*p* ligands play a significant role. Ni^III^ oxides such as *R*NiO_3_, *R*SrNiO_4_ (*R* = rare earth element), and LiNiO_2_ are in the negative charge-transfer energy regime (Δ < 0, Fig. 1a), leading to 3*d*^8^*L* (*L* denotes a ligand hole located on a molecular orbital formed by the O 2*p* orbitals) as the dominant ground state configuration, instead of the conventional 3*d*^7^ (Δ > 0, Fig. 1b)^14–19^. It is well known that as the Ni^III^ oxidized to Ni^IV^, the electron removal in such charge transfer system should happen in the O 2*p* band rather than the Ni 3*d* band^20^, resulting in a 3*d* ^8^*L*^2^ configuration, namely double O 2*p* hole states (Fig. 1a).

Based on the lattice oxygen oxidation mechanism, we proposed that a catalyst featuring double ligand hole states would endow high OER activity. To test our prediction, LiNiO_2_ is employed as the precatalyst for the double ligand hole states considering that it has an edge-shared Ni-O-Ni network, which is more stable than the corner-shared network in *R*NiO_3_^21^ and *R*SrNiO_4_^22^. In addition, as a well-known cathode material of LIB, LiNiO_2_ can be easily delithiated and oxidized to form Ni^IV^ ions^23,24^, although this oxidizing process is rarely observed during OER in aqueous electrolytes. The stabilization of the highly active Ni^IV^ has been recently attributed to the high-entropy charge-glass-like state of LiNiO_2_^17^. The electronic structure of LiNiO_2_ is found to have many nearly degenerate states composed of [NiO_6_] octahedra with 3*d*^8^, 3*d*^8^*L* and 3*d*^8^*L*^*2*^ configurations^17^. The LiNiO_2_ system can fluctuate between these states such that the hole states on the oxygen ligands are stabilized by the entropy term of the free energy.

In this study, a pristine Ni^2+^-free LiNiO_2_ sample was prepared using high-pressure O_2_, and its structure was comprehensively characterized using multiple complementary techniques, including microscopic techniques, X-ray diffraction (XRD), and X-ray absorption spectroscopy (XAS) at the Ni *K*, Ni *L*_3,2_, and O *K* edges. The electrocatalytic properties of LiNiO_2_ was robustly assessed using rotating ring disk electrodes (RRDE). The delithiation, oxidation and resulting structural transition of LiNiO_2_ during OER were probed using operando XRD, XAS and Raman spectroscopies. Combined analysis of the operando results, density function theory (DFT) calculation and in situ differential electrochemical mass spectrometry (DEMS) reveals that the enhanced OER activity of LiNiO_2_ is originated from the structurally stabilized Ni^IV^ with a 3*d*^8^*L*^2^ configuration.

## Results

### Structural characterization of LiNiO_2_

**Fig. 2:**
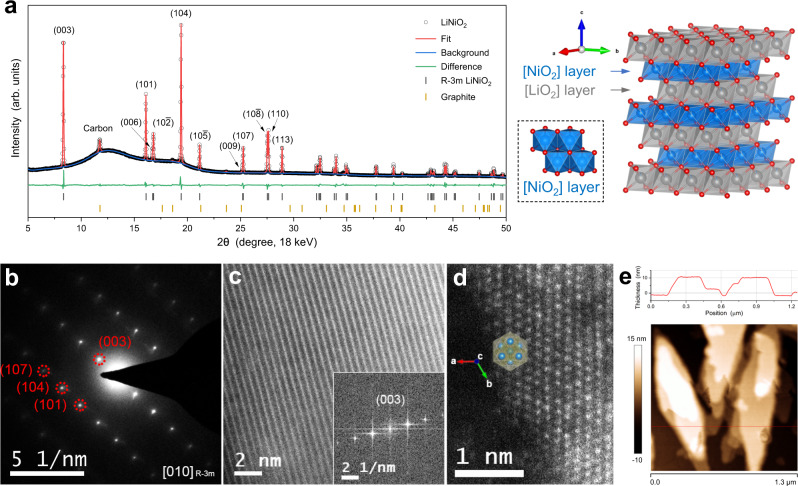
Structural characterization of the as-prepared LiNiO_2_. **a** A synchrotron XRD pattern (18 keV incident beam energy, λ = ~0.6888 Å) and a Rietveld refinement fit with the refined structural parameters listed in Supplementary Table 1, along with the structural model of R-3m LiNiO_2_ which consists of alternate [LiO_2_] and [NiO_2_] layers formed by edge-sharing [LiO_6_] or [NiO_6_] units. A set of extra diffraction peaks are from graphitic carbon of carbon paper and two broad diffraction peaks at 12° and 18° are from the sample holder. **b** A typical SAED pattern of LiNiO_2_ along a [010] zone axis of the R-3m structure, **c** a HRTEM image and its fast Fourier transform pattern, showing the (003) lattice fringes, and **d** an AC-HAADF-STEM image projected perpendicular to the (003) lattice plane. **e** An AFM image and the corresponding thickness profile across the horizontal red line.

The crystalline phase and morphology of the as-prepared LiNiO_2_ were characterized using synchrotron X-ray diffraction (XRD) and transmission electron microscopy (TEM) (Fig. 2). The XRD pattern of LiNiO_2_ is well-matched with the standard pattern of R-3m LiNiO_2_ (JCPDS No. 09-0063) without any noticeable crystalline impurities (Fig. 2a and Supplementary Fig. 1). The sharp diffraction peaks, and the well separation of (006) and (10-2) and of (10-8) and (110), indicate a well-crystalized layered structure with a low degree of Li^+^/Ni^2+^ intermixing (Supplementary Fig. 1)^23,25^. Rietveld refinement was carried out to obtain the crystal structural parameters of the as-prepared LiNiO_2_ (Fig. 2a), yielding a good fit for the R-3m structure and ~3.9% Ni occupancy in the [LiO_2_] layers, demonstrating the high-purity of the sample. The single crystalline nature of the as-prepared LiNiO_2_ is suggested by TEM techniques (Fig. 2b–d). A typical selected-area electron diffraction (SAED) pattern of LiNiO_2_ shows clear diffraction spots that can be indexed to the R-3m structure of LiNiO_2_ along the [010] zone axis (Fig. 2b). High-resolution TEM (HRTEM) images of the thin parts of LiNiO_2_ display uniform lattice fringes of (003) and (104) of the R-3m structure, respectively (Fig. 2c and Supplementary Fig. 2). Aberration-corrected high-angle annular dark-field scanning transmission electron microscopy (HAADF-STEM) images were also recorded to directly visualize the Ni atomic arrangement along the edges, which well matches the (003) lattice plane of R-3m LiNiO_2_ (Fig. 2d). TEM, scanning electron microscopy (SEM) and atomic force microscopy (AFM) suggest that the general morphology of the as-prepared LiNiO_2_ is nanoflakes with 200 − 400 nm in the lateral size and ~10 nm in the layer thickness (Supplementary Fig. 3 and Fig. 2e). LiNiO_2_-raw, a LiNiO_2_ sample prepared without the second annealing step under high-pressure O_2_, shares a similar morphology with the LiNiO_2_ (Supplementary Fig. 4).

**Fig. 3:**
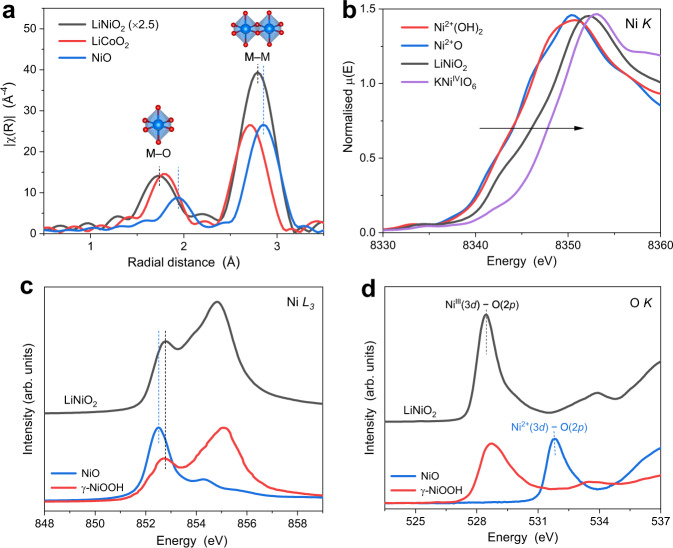
XAS characterisation of the as-prepared LiNiO_2_. **a** Fourier transforms of *k*^3^-weigted EXAFS spectra of LiNiO_2_, LiCoO_2_ (R-3m) and NiO, and **b** XANES spectra of LiNiO_2_ and Ni references (NiO, Ni(OH)_2_, and KNiIO_6_) at the Ni *K* edge. The amplitude of LiNiO_2_ EXAFS spectra is adjusted for a clear comparison, and the Fourier transforms were phase-corrected. TEY-XAS spectra of LiNiO_2_ **c** at the Ni *L*_3_ edge and **d** at the O *K* edge, along with NiO and γ-NiOOH as the Ni and O references.

The local coordination environment of transition metal ions can be obtained by extended X-ray absorption fine structure (EXAFS) spectra at the corresponding *K* edges^26,27^. The Ni local coordination of LiNiO_2_ resemble that of NiO and Ni(OH)_2_, with two dominant peaks at ~1.8 and ~2.8 Å, which are attributed to Ni–O and Ni–Ni scattering paths within and between the edge-sharing [NiO_6_] units, respectively (Fig. 3a and Supplementary Fig. 5). For these peaks, LiNiO_2_ shows a shorter radial distance than those of Ni(OH)_2_ and NiO, supporting the presence of a shorter Ni-O bond length due to the higher formal oxidation state of Ni.

From an ionic picture, low spin Ni^III^ ions adopt a (*t*_2*g*_)^6^(*e*_*g*_)^1^ configuration in an octahedral crystal field and are Jahn-Teller (JT)-active, so that one can expect a strong local distortion of the [NiO_6_] octahedra to remove the degeneracy of the *e*_*g*_ orbitals. There are local distortions of the [Ni^III^O_6_] units here, as evidenced by the broader first coordination shell at 1.7 A and a larger disorder factor (σ^2^) of LiNiO_2_ by fitting *R*-space EXAFS spectra, as compared with that of the analogous R-3m LiCoO_2_ (Co^III^, (*t*_2*g*_)^6^(*e*_*g*_)^0^, Fig. 3a, Supplementary Fig. 6, and Table 1). Two distortion mechanisms are proposed. One is the conventional local JT distortion, as suggested by previous EXAFS studies of LiNiO_2_^28,29^, with four short Ni-O (~1.920 Å) and two long Ni-O (~2.069 Å) bonds in the [Ni^III^O_6_] octahedra (Table 1 and Supplementary Fig. 7a). However, pure JT distortion fails to explain the neutron-pair distribution function and electronic structure of LiNiO_2_; therefore, a [NiO_6_]-size disproportionation (SD) mechanism was proposed, where the number of Ni−O_short_ and Ni−O_long_ is equal^17^ (Table 1 and Supplementary Fig. 7b). Based on these two possible structural models, EXAFS fitting using two Ni−O coordination shells was performed. Both models gave comparable but better fits of the EXAFS data than that using only one Ni–O scattering path (Table 1 and Supplementary Fig. 7c), supporting the presence of distorted [Ni^III^O_6_] in pristine LiNiO_2_. To distinguish JT distortion from SD, double cluster simulation would be needed to simulate the Ni-*L*_2,3_ XAS spectrum of LiNiO_2_^16^, which is beyond the scope of this study.

**Table 1:** Structural parameters of LiNiO_2_, LiCoO_2_, and NiO, obtained from EXAFS fitting^a^

Sample	Scattering path^b^	*R* (Å)	*N*	*σ*^*2*^ (x10^-3^ Å^2^)	R factor (%)
LiNiO_2_ (JT)	Ni–O_short_	1.919 ± 0.006	4^c^	5.4 ± 0.7	0.28
	Ni–O_long_	2.071 ± 0.014	2^c^	5.6 ± 1.8	
	Ni–Ni	2.886 ± 0.003	5.7 ± 0.4	5.0 ± 0.4	
LiNiO_2_ (SD)	Ni–O_short_	1.905 ± 0.008	3^d^	4.5 ± 0.9	0.26
	Ni–O_long_	2.040 ± 0.012	3^d^	8.1 ± 2.2	
	Ni–Ni	2.886 ± 0.003	5.7 ± 0.4	5.0 ± 0.3	
LiNiO_2_^e^	Ni–O	1.948 ± 0.009	5.2 ± 1.1	9.1 ± 2.1	0.82
	Ni–Ni	2.884 ± 0.005	5.6 ± 0.6	4.9 ± 0.5	
NiO	Ni–O	2.077 ± 0.005	5.7 ± 0.7	5.8 ± 1.0	0.29
	Ni–Ni	2.950 ± 0.003	11.2 ± 0.6	5.9 ± 0.3	
LiCoO_2_	Co–O	1.915 ± 0.007	5.8 ± 0.4	2.8 ± 0.7	0.43
	Co–Co	2.812 ± 0.004	6.4 ± 0.4	3.0 ± 0.4	
LiNiO_2_^f^	Ni–O	1.976	6	N.A.	N.A.
	Ni–Ni	2.883	6		

Plots and the corresponding fits are shown in Supplementary Fig. 7 and Supplementary Fig. 8.

^a^*R* is the distance between the absorber–scatterer pair, *N* is the coordination number, *σ*^*2*^ is the Debye-Waller (disorder factor), and R factor is a measure of the goodness of fit.

^b^A Ni–Li scattering path (Reff=2.893 Å) was not included in the fits of LiNiO_2_, because of the low scattering amplitude of Li^+^ and because Ni–Li has no significant effect on the R-factor and the obtained parameters.

^c^not allowed to vary considering the Jahn-Teller (JT) distortion of Ni^III^. If both N(Ni–O_short_) and N(Ni–O_long_) are allowed to vary, 10 variables out of 14 independent points are required and the fit yield a negative *σ*^*2*^(Ni–O_short_) and a relatively large *σ*^*2*^(Ni–O_long_).

^d^not allowed to vary considering that a size-disproportionated (SD) [NiO_6_] model, where N(Ni–O_short_) = N(Ni–O_long_)=3 (Ref. ^17^).

^e^In this fitting model, the distortion of Ni-O bonds is considered in σ^2^(Ni–O).

^f^calculated from R-3m LiNiO_2_ crystal structure (ICSD collection code: 78687) using Feff6.

The oxidation state of the as-prepared LiNiO_2_ was assessed by X-ray absorption near edge structure (XANES) spectroscopy (Fig. 3b). At the Ni *K* edge, the energy position of LiNiO_2_ locates in-between those of Ni^2+^ (NiO and Ni(OH)_2_) and Ni^IV^ references (KNiIO_6_), suggesting that the Ni of the LiNiO_2_ is in the intermediate oxidation state between Ni^2+^ and Ni^IV^. Assuming that the formal oxidation state of Ni is proportional to the energy at 0.7 edge jump height, the average Ni oxidation state of LiNiO_2_ can be quantified as ~3.0 (Supplementary Fig. 9), in a good agreement with the nominal value.

The detailed electronic structure at the surface region can be obtained by total electron yield soft X-ray absorption spectra (TEY-sXAS) at the *L*_*3*_ edges. The multiplet spectral feature and the energy position at the *L*_*3*_ edges of 3*d* elements are highly sensitive valence state and local environments^30–33^. Figure 3c shows the Ni *L*_*3*_ edge sXAS of LiNiO_2_, along with those of NiO and γ-NiOOH as the Ni^2+^ and Ni^III^ references, respectively. Both the multiplet spectral features and energy positions of the Ni-*L*_*3*_ edge are very similar to those of *γ-*NiOOH, indicating that the Ni of LiNiO_2_ is in the formal +3 oxidation state. The dominant peak for both γ-NiOOH and LiNiO_2_ locates at ~855 eV, much higher than that of NiO at ~853 eV, and further inspection reveals that the low-energy peak of LiNiO_2_ locates ~0.3 eV higher than the dominant peak of NiO in Fig. 3c. The low-energy peak is an intrinsic spectral feature of Ni^III^ as found in theoretical and experimental XAS^15,16^ and high-quality single crystal LaNiO_3_ and NdSrNiO_4_^21,22^ In addition, Ni^III^ (3*d*^8^*L*) in our LiNiO_2_ is supported by theoretical simulation of the Ni *L*_3,2_ XAS in Supplementary Fig. 10a, where the LiNiO_2_ spectra can be nicely reproduced by the full multiplet cluster calculation with a nominal 3*d*^8^*L* configuration. Our LiNiO_2_ is free from Ni^2+^ impurity that often observed in the literature (Supplementary Fig. 10b).

The Ni valence state and Ni-O covalency were further studied by the O*-K* sXAS. The pre-edge peak below 533 eV can be assigned to the unoccupied 3*d e*_*g*_ hybridised with O 2*p* orbitals. An increase in the valence state of the 3*d* element correlates with a shift of the pre-edge to lower energy and an enhancement of the spectral intensity due to the strong covalence^21,34,35^. The O-*K* sXAS of LiNiO_2_ is dominated by a strong peak at 528.2 eV without obvious contribution of a peak at 531.9 eV found from NiO (Fig. 3d). Thus, TEY-sXAS at both the Ni *L*_*3*_ edges and the O *K* edge suggest that the surface Ni oxidation state of LiNiO_2_ is Ni^III^ and free from Ni^2+^ impurities.

### Electrochemical measurements

**Fig. 4:**
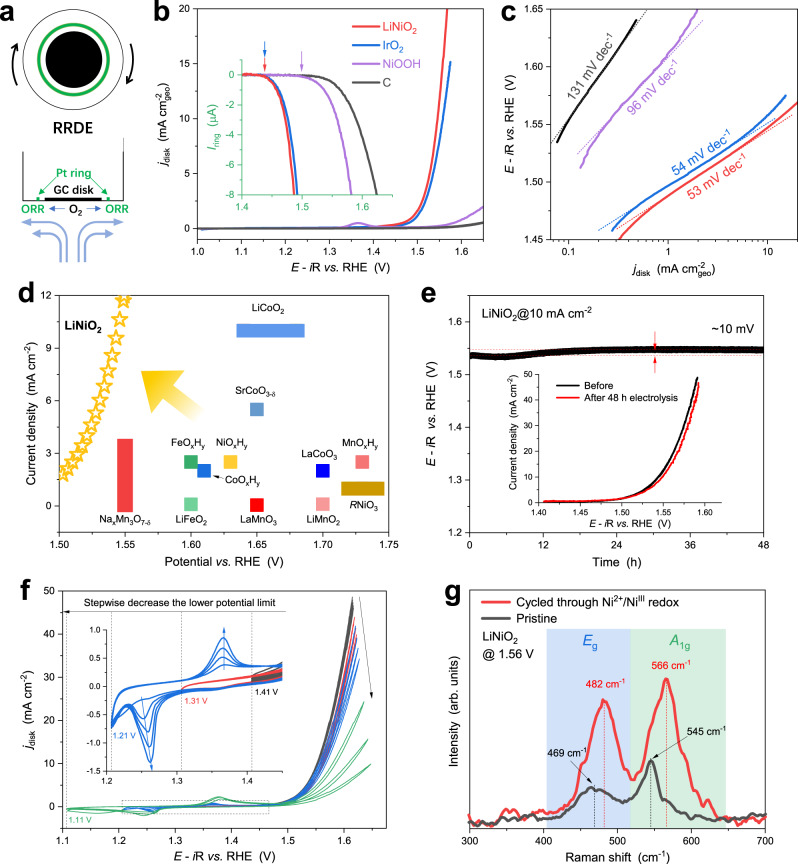
Electrochemical measurements and surface chemistry of LiNiO_2_. **a** Schematic illustration of a rotating ring disk electrode (RRDE, ring: Pt, disk: glassy carbon). **b** Linear sweep voltammograms and **c** Tafel plots of LiNiO_2_, NiO-derived NiOOH, IrO_2_ (99.9% metal basis, Adamas-beta) and carbon (Vulcan XC-72R) in Ar-saturated 1 M KOH solution, along with the corresponding Pt ring current shown in the inset (the onset potential of OER indicated by arrows). **d** OER activity comparison of the as-prepared LiNiO_2_ to LiMO_2_ (M = Co and Fe), and *R*MO_3_ (*R* = Rare earth elements, M = Fe, Co and Ni) and other unary 3d transition metal catalysts (Supplementary Table 3). **e** Stability test of LiNiO_2_ in 1 M KOH at 10 mA cm^−2^ (on carbon paper) and (the inset of **e**) linear sweep voltammograms of LiNiO_2_ before and after the stability test. **f** Window-opening cyclic voltammograms of LiNiO_2_ in Fe-free 1 M KOH with stepwise decreasing the lower potential limits, showing the evolution of redox peaks (magnified in the corresponding insets) and the deterioration of OER current. The voltammograms were collected under 1600 rpm with a sweep rate of 5 mV s^−1^, and IR drops of the voltammograms were determined by EIS prior to the measurements and compensated. **g** Operando Raman spectra of LiNiO_2_ at 1.56 V with and without being cycled through Ni^2+^/Ni^III^ redox.

The OER activity of LiNiO_2_ was studied using rotating ring disk electrodes (RRDE, disk: glassy carbon, ring: Pt) at 1600 rpm. The potential of the Pt ring was held at 0.42 V throughout the measurements, so that O_2_ once generated on the disk can be collected and electrochemically reduced on the ring (Fig. 4a and Supplementary Fig. 11), which enables the determination of OER onset potentials. The OER activity of LiNiO_2_ was estimated using linear sweep voltammetry (LSV) in Ar-saturated and Fe-free 1 M KOH and compared with the benchmark IrO_2_ (99.9%) catalyst and a NiOOH reference. The LiNiO_2_ shows the best OER geometric activity (Fig. 4b, current density normalized by disk electrode area) and intrinsic activity (Supplementary Fig. 13, current density normalized by electrochemical surface area), with the potential to achieve 10 mA cm_geo_^−2^ (E@10 mA cm_geo_^−2^, ~1.545 V) lower than those of IrO_2_ (~1.558 V) and NiOOH (~1.753 V). In addition, LiNiO_2_ shares a Tafel slope (53 mV dec^−1^) similar to IrO_2_ (54 mV dec^−1^) and distinguishes itself from conventional NiOOH (96 mV dec^−1^, Fig. 4c), indicating rapid OER kinetics of LiNiO_2_. Compared to the reported overpotential values of LiMO_2_ (M = Co and Fe), and *R*MO_3_ (*R* = Rare earth elements, M = Fe, Co and Ni) and other unary 3*d* transition metal catalysts, our LiNiO_2_ requires much less overpotential to achieve certain current density (Fig. 4d), demonstrating the unique electrochemical properties to facilitate OER. Amongst the previously reported LiNiO_2_ and even Fe-doped LiNiO_2_, the as-prepared sample exhibit superior E@10 mA cm^−2^ and Tafel slopes, highlighting the excellent activity of pristine LiNiO_2_ (Supplementary Table 2).

The stoichiometric structure of LiNiO_2_ is essential to achieve high OER activity. LiNiO_2_-raw was also characterized and compared with the pristine LiNiO_2_, as the high-pressure or high-flow-rate O_2_ during synthesis has been found to be essential for stabilizing Ni^III^ and minimizing oxygen vacancies in LiNiO_2_ and LiNiO_2_-based cathode materials^36,37^. The LiNiO_2_-raw shares similar morphology with the LiNiO_2_ (Supplementary Fig. 4) but has ~4.6% oxygen vacancies (*vs*. ~0.4% in LiNiO_2_) as characterized by XRD refinement (Supplementary Fig. 14 and Supplementary Table 4), and a significant fraction of Ni^2+^ impurities as shown in Ni-*K* XAS (Supplementary Fig. 15) and surface sensitive TEY-sXAS at the Ni *L*_3_ and O *K* edges (Supplementary Fig. 16 and 17). We found that the off-stoichiometric structure of LiNiO_2_-raw yields reduced OER activity (Supplementary Fig. 18).

From the ring current of the RRDE, the OER onset potentials of LiNiO_2_, IrO_2_ and NiOOH were estimated as ~1.437, ~1.438 and ~1.495 V, respectively (Fig. 4b and Supplementary Fig. 11), indicating that LiNiO_2_ activates the oxidation of water at a much lower overpotential than NiOOH. The OER activity of NiOOH is consistent with a common understanding that Ni (oxy)hydroxides without Fe doping and without the presence of Fe in the electrolyte are poor OER electrocatalysts^38–40^. Thus, from perspectives of overpotentials, Tafel slopes and onset potentials, the electrocatalytic properties of LiNiO_2_ for OER is as good as the state-of-the-art IrO_2_ and outperforms the standard NiOOH. The stability of LiNiO_2_ was measured using chronopotentiometry at 10 mA cm^−2^ (Fig. 4e). LiNiO_2_ largely maintained its initial potential, which gradually increased by ~10 mV for the first 18 h, and then remained constant for the subsequent 30 h. The high stability of LiNiO_2_ is also indicated by the little activity deterioration in LSV before and after the 48 h stability test (the inset of Fig. 4e). One may be interested in the stability of LiNiO_2_ as an OER electrocatalyst, as LiNiO_2_ is known to suffer from severe capacity degradation as a cathode material for LIB. The fast capacity fading is generally observed in high voltage region >4.1 V *vs*. Li^+^/Li, resulting from a large content of delithiation in Li_x_NiO_2_ (x < 0.3) and in turn structural collapse^23,41,42^. If the highest cut-off voltage would be limited to 4.1 V *vs*. Li^+^/Li, the capacity of LiNiO_2_ could retain >95% of its initial capacity after >100 cycles^41,43^. However, operation potentials for our OER are below 1.8 V *vs*. RHE which corresponds to only 4.03 V *vs*. Li^+^/Li in battery operation.

The interconversion of Ni^2+^-based (hydr)oxides to NiOOH is usually indicated by Ni^2+^/Ni^III^ redox peaks in the potential window of 1.2–1.4 V (*vs*. RHE); therefore, the formation of NiOOH from LiNiO_2_ was investigated using window-opening cyclic voltammetry. As shown in Fig. 4f, the lower potential limit decreased stepwise from 1.41 V (used in Figs. 4a) to 1.11 V, close to the values generally used in previous studies^44–49^. When the lower potential limit was set to 1.41 V, the OER current is optimal and remains stable over multiple CV cycles. However, decreasing the limit to ~1.21 and ~1.11 V deteriorates the OER current significantly, accompanied by the appearance and gradual increase of redox peaks at ~1.36 (anodic) and ~1.26 V (cathodic), indicating the formation of redox-active but OER-inactive phases on the surface. A LiNiO_2_ sample, cycled through the Ni^2+^/Ni^III^ redox, was characterized using operando Raman spectroscopy at 1.56 V (Fig. 4g). Its Raman spectrum shows clear differences in the position and relative intensity of the Raman bands and a considerable increase in the overall Raman scattering intensity, as compared to pristine LiNiO_2_ at the same potential (for more details, see the operando Raman section below). All these spectral features can be indexed to β-NiOOH^50^, indicating the structural transformation of OER-inactive NiOOH species from LiNiO_2_ after being cycled through Ni^2+^/Ni^III^ redox. Window-opening voltammetry was also carried out in unpurified 1 M KOH (Supplementary Fig. 19), wherein, despite the appearance of the Ni^2+^/Ni^III^ redox peaks in the close potential range, the OER current remains stable even when the lower potential limit reached ~1.11 V. This indicates that Fe impurities significantly improve the performance of NiOOH, in agreement with the common understanding of Ni-based OER electrocatlaysts^38–40^. Therefore, the voltammetric analysis shows that the OER activity and electrochemical properties of LiNiO_2_ are impacted by the lower potential limits and presence of Fe impurities in the electrolyte. When undergoing the Ni^2+^/Ni^III^ redox, the surface structure of LiNiO_2_ transforms and degrades into NiOOH, which has a poor OER activity in the absence of Fe. In contrast, the pristine and structurally unaltered LiNiO_2_, whose real active species has been covered and neglected in previous LiNiO_2_ OER studies^44,45,47–49^, exhibits better intrinsic OER activity.

### Operando XRD, XAS, and Raman studies

From the aforementioned structural and electrochemical measurements, the pristine nature of LiNiO_2_ has been characterised in terms of crystalline phase, local coordination environment, oxidation state and electronic structure, and the superior OER activity of LiNiO_2_ to the analogous NiOOH is confirmed and studied in detail. However, the active species of LiNiO_2_, which can no longer be attributed to NiOOH, remains unclear. In addition, LiNiO_2_, a typical cathode material for lithium-ion batteries, can be delithiated to increase the Ni oxidation state and induce structural transition;^24,46^ therefore, delithiation is expected to become comparable or even easier during the OER in KOH aqueous solution. To establish the relationship between the structure of LiNiO_2_ and its OER activity, operando synchrotron-based XRD and XAS, and Raman spectroscopy experiments were carried out.

**Fig. 5:**
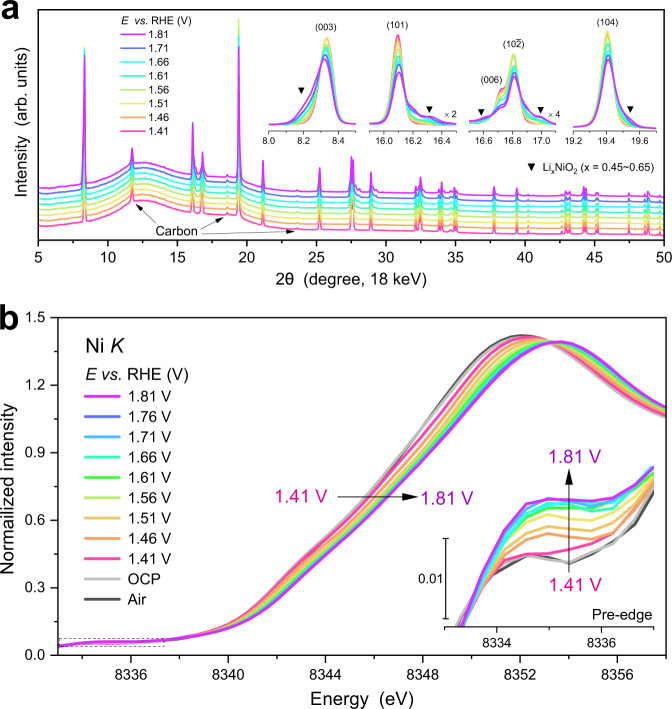
LiNiO_2_ delithiation under OER conditions. **a** Operando XRD patterns and **b** Operando Ni *K* edge XANES spectra of LiNiO_2_ under applied potentials of 1.41–1.81 V in 1 M KOH solution. Diffraction peaks in low 2θ ranges and the pre-edge peak were magnified in the corresponding insets. A set of extra peaks are from graphitic carbon and broad diffraction peaks at 12° and 18° from the sample holder.

Figure 5a shows the operando synchrotron XRD of LiNiO_2_ in 1 M KOH in the potential range of 1.41–1.81 V. The patterns consist of three parts: an amorphous background from cell body and window materials (~12° and 18°), diffraction peaks of graphitic carbon from carbon paper (indicated by arrows), and diffraction patterns of LiNiO_2_ and its delithiated derivatives. Using the carbon peak at ~11.8° as the internal reference for intensity and peak position (Supplementary Fig. 20), the overall LiNiO_2_ pattern largely remains intact under the applied potentials, and another set of pattern emerges with potentials in around 2θ values at the expense of LiNiO_2_ pattern intensity when the applied potential is greater than 1.46 V, coinciding with the onset of OER. Whilst (00 l) diffraction peaks of LiNiO_2_ show shoulders at lower 2θ values, the (h0l) peaks show shoulders at higher 2θ values, which suggests the formation of another hexagonal phase from the parent LiNiO_2_ with interlayer expansion and intralayer contraction for the [NiO_2_] layers^24^. Based on the peak position, the new phase can be indexed to layered Li_x_NiO_2_ (x = 0.45–0.65, Supplementary Fig. 21)^51^, and other Li_x_NiO_2_ polymorphs and NiOOH phases were not found. The LiNiO_2_ delithiation during OER is also supported by increased Li concentration of KOH electrolyte and Ni/Li molar ratio of the sample (Supplementary Table 5). Considering that both the delithiation and OER initiate from the surface, the delithiated Li_x_NiO_2_ can be considered as the active species of OER.

**Fig. 6:**
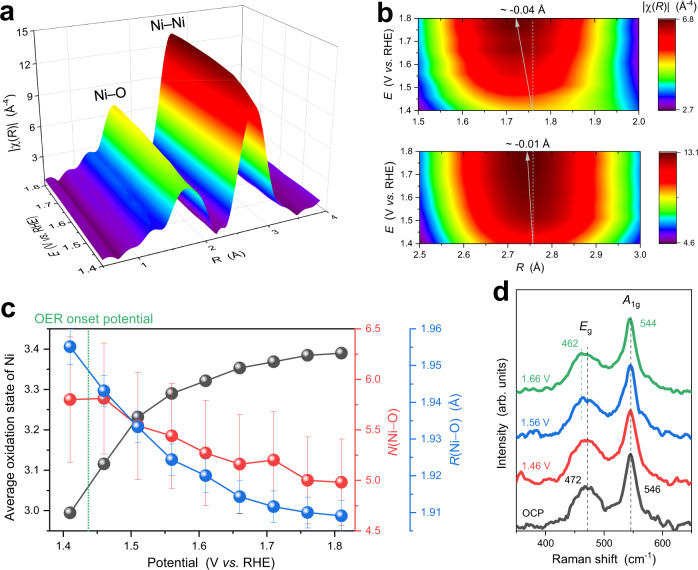
Local coordination of LiNiO_2_ under OER conditions. **a**, **b** Fourier transforms of operando Ni *K* edge EXAFS spectra of LiNiO_2_ under applied potentials of 1.41–1.81 V in 1 M KOH solution, with the Ni–O region and the Ni–Ni region magnified to better show the potential-dependent behaviour. The Fourier transformation was carried out over a k range of 2.5 − 12.4 Å^−1^, and the Fourier transforms were phase-corrected. **c** Average oxidation state, *N*(Ni–O) and *R*(Ni–O) of LiNiO_2_ as a function of applied potentials. The error bars represent the uncertainty of the fitted parameters. **d** Operando Raman spectra of LiNiO_2_, showing the *E*_g_ and *A*_1g_ vibration bands, where oxygen atoms of [NiO_6_] units move oppositely along adjacent O-layers and symmetrically along the c-axis, respectively.

The oxidation of LiNiO_2_ accompanying with delithiation was tracked using operando XANES measurements at the Ni *K* edge. No discernible change in XANES spectra was observed when the electrode was immersed in 1 M KOH (Supplementary Fig. 22). Figure 5b shows the XANES spectra in the potential range of 1.41–1.81 V. The rising edge shifts towards higher energy with the applied potentials, indicating that the oxidation of Ni^III^ coincides with the onset of OER. Using the relationship between the energy at 0.7 edge jump height and the Ni formal oxidation state obtained from ex-situ data (Supplementary Fig. 9), the average Ni oxidation state of LiNiO_2_ was plotted as a function of the applied potentials (Fig. 6c). The oxidation state is ~3.0 at 1.41 V, sharply increases to ~3.3 at 1.56 V, gradually at potentials >1.56 V and reaches a plateau of ~3.4. In addition, the pre-edge peak intensity was found to increase with applied potentials (the inset of Fig. 5b), corresponding to the decreased symmetry of [NiO_6_] octahedra (see EXAFS analysis below). Combining the potential-dependent oxidation state of Ni with the electrochemistry of LiNiO_2_ reveals a positive correlation between the number of Ni^IV^ ions and OER current (Fig. 6c), suggesting that Ni^IV^ in delithiated Li_x_NiO_2_ can be considered as (part of) the OER active centre.

The dominance of Ni^IV^ with double ligand hole state (3*d*^8^*L*^2^) is supported by simulation of the experimental sXAS at the Ni-*L*_3,2_ edge of LiNiO_2_ after OER taken with surface sensitive TEY mode (black circles) using the full multiplet cluster calculation which includes full intra-atomic multiplet interaction, crystal field interaction and covalence. We found the experimental Ni-*L*_3,2_ sXAS of LiNiO_2_ after OER can be well reproduced by the sum (black line) of 75% 3*d*^8^*L*^2^ (blue line) and 25% 3*d*^8^*L* (red line) configurations (Supplementary Fig. 23b) giving rise to Ni^3.75+^ valence state.

Potential-dependent changes of LiNiO_2_-raw in XANES are much weaker than those of pure LiNiO_2_ (Supplementary Fig. 24). The average oxidation state of Ni of LiNiO_2_-raw grows much slower with applied potentials than that of LiNiO_2_, and reaches only ~3.2 at 1.81 V. The impeded Ni oxidation in LiNiO_2_-raw implies that the formation of Ni^IV^ and double ligand hole state in LiNiO_2_-raw is limited. For the LiNiO_2_-raw sample, the XRD patterns show increasing fraction of NiOOH phase with applied potentials, while the R-3m structures is largely maintained in LiNiO_2_ without discernible formation of any NiOOH phases (Supplementary Fig. 25). It should be noted that the nanoscale thickness of our LiNiO_2_ is a prerequisite to observe significant spectral changes in operando XRD and operando XAS, because the OER usually occurs within several nanometres from the surface. Thus, our results indicates that observation of Ni^IV^ with double ligand holes state is directly related to both our pure and nano-sheet pristine LiNiO_2_ sample.

Potential-dependent changes in the local coordination environment of Ni were probed using operando EXAFS (Fig. 6a, b). Similar to the ex-situ data, the Ni coordination shell is dominated by Ni–O and Ni–Ni scattering paths. The amplitude of Ni–O at ~1.45 Å increases with the applied potentials from 1.41 to 1.81 V (Fig. 6b). Because the Ni first-shell coordination in pristine LiNiO_2_ is saturated with six oxygen atoms, the coordination numbers (CN) of Ni-O cannot increase further, which alternatively implies the Ni-O bonds become increasingly ordered with the applied potentials. The lowered Ni-O distortion is also suggested by the decreased Ni-O peak border at the high *R* with increased amplitude (Fig. 6b) and by the increased frequency of Ni-O oscillation in the real part of the Fourier transforms (Supplementary Fig. 26). Quantitative structural information was extracted via EXAFS fitting (Supplementary Fig. 27 and Supplementary Table 6), where the parameter σ^2^(Ni–O) represents the distortion in the [NiO_6_] octahedra. The value of σ^2^(Ni–O) decreases as a function of the applied potential from 0.012 Å^−2^ at 1.41 V to 0.008 Å^−2^ at 1.81 V, supporting that the LiNiO_2_ evolves from the oxidation of SD-active Ni^III^ atoms (t_2g_^6^e_g_^1^) to SD-inactive Ni^IV^ atoms (t_2g_^6^) under OER. In addition, the CN(Ni–O) decreases from 5.8 ± 0.6 at 1.46 V to 5.0 ± 0.4 at 1.81 V, indicating the formation of oxygen vacancies during OER^52–54^, which have been calculated to greatly reduce the OER overpotential by enhancing intermediate adsorption on the Ni sites^53,54^. In addition, the increase in the relative spectral intensity of the weak pre-edge peak at 8335.4 eV in the Ni-*K* XANES as a function of applied potentials (the inset of Fig. 5b) implies a reduced local symmetry of Ni ion, which generally suggests a decrease in CN^53^. Moreover, our DEMS measurements and DFT calculation indicate that lattice oxygen involves in OER process (see below). In this lattice oxygen mechanism, oxygen vacancies should be created.

In addition to the amplitude, the contour maps of Ni–O shows a gradually increasing contribution in the corresponding lower *R* regions (Fig. 6b), corresponding to a shift toward lower *R* in the real part of FT-EXAFS (Supplementary Fig. 26). A shrinking Ni–O bond length from 1.955 Å at 1.41 V to 1.909 Å at 1.81 V was determined via EXAFS fitting (Fig. 6c, Supplementary Fig. 27 and Supplementary Table 6), supporting the presence of a high valence state of Ni^IV^ under OER conditions. To summarise, by combining the fast operando Ni-*K* XANES and EXAFS spectra with the OER onset potential, Ni^IV^ and oxygen vacancies in delithiated Li_x_NiO_2_ were identified during OER, and attributed to the high OER activity.

Operando Raman spectroscopy was further employed to track the intermediate species of LiNiO_2_ under the high applied potentials larger than 1.4 V. Different from the above Raman experiments in Fig. 4g, the LiNiO_2_ now was not forced to undergo Ni^2+^/Ni^III^ redox, but directly applied to 1.46 V. The spectral profile of LiNiO_2_ Raman in Fig. 6d only exhibits slightly red-shifted bands (*E*_g_: from 472 cm^−1^ to 462 cm^−1^; *A*_1g_: from 546 cm^−1^ to 544 cm^−1^) with increasing applied potentials during the OER process, in agreement with the formation of delithiated LiNiO_2_^55^. This supports the results from the above operando XRD and XAS. From comparison between Fig. 6d and Fig. 4g we can conclude that the crystal structure, intermediates and the OER activity (Fig. 4f) of LiNiO_2_ strongly depend on the lowest potential applied.

The presence and formation of γ-NiOOH have been regarded as the origin of OER activity in many Ni-based oxides^46,53,56^. However, according to our operando Raman spectra (Fig. 6d), the active species of LiNiO_2_ could not be assigned to γ-NiOOH as per the band position and relative intensity (for LiNiO_2_, *A*_1g_: ~546 cm^−1^ and I(*E*_g_)/I(*A*_1g_)=0.5; for γ-NiOOH, *A*_1g_: 558 − 560 cm^−1^ and I(*E*_g_)/I(*A*_1g_) = 1.5 − 1.7)^40,57–59^. In addition, a large expansion in the interlayer spacing between [NiO_2_] layers, caused by the transformation from LiNiO_2_ (4.72 Å) to γ-NiOOH (≥7 Å), was not observed in our operando XRD data. In addition, post-mortem XRD and XAS indicate that the structure of Li_x_NiO_2_ remains mainly intact after OER (Supplementary Fig. 29 and 30). Moreover, the surface and bulk AC-STEM images were employed to study the structural transformation of LiNiO_2_ after OER^60,61^ (Supplementary Fig. 31), and both show the atomic arrangement perpendicular to the (018) lattice plane of LiNiO_2_, supporting the surface structure of LiNiO_2_ remains mainly intact after OER. Thus, using operando X-ray diffraction/absorption and Raman spectroscopies and post-mortem characterizations, the active species were identified as the delithiated yet structurally intact LiNiO_2_ with oxygen vacancies.

### DFT calculation and DEMS for OER mechanisms

**Fig. 7:**
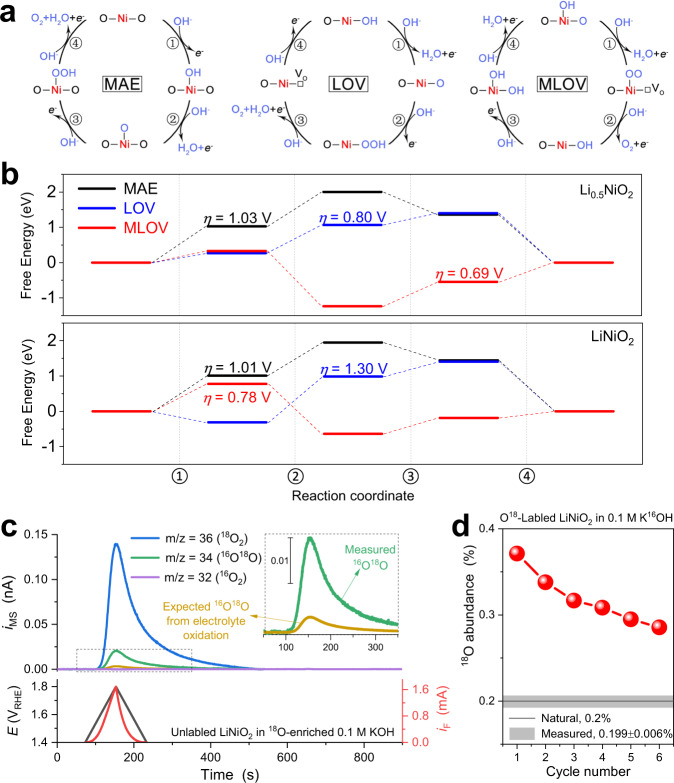
The OER mechanism of LiNiO_2_. **a** Schematic 4-step reaction pathways of MAE, LOV, and MLOV OER mechanisms. Free energy of each OER step of **b** Li_0.5_NiO_2_ and LiNiO_2_ at U_RHE_ = 1.23 V. The estimated thermodynamic overpotentials of each potential-determining step are labelled. **c** MSCV and faradic CV curves recorded in ^18^O-enriched 0.1 M KOH electrolyte prepared using H_2_^18^O (99% ^18^O), showing the mass ion current (*i*_MS_) of ^18^O_2_, ^16^O^18^O and ^16^O_2_ along with the faradaic current (*i*_F_) and the applied potential *E* from the first CV scan. (The inset of **c**) Comparison of the measured *i*_MS_(^16^O^18^O) curve and the theoretically expected *i*_MS_(^16^O^18^O) curve purely from electrolyte oxidation, based on the ^16^O abundance of 1.18% in the electrolyte (Supplementary Equation S27). **d** The evolution of measured ^18^O abundance of evolved oxygen (from total DEMS charge) for ^18^O-labled LiNiO_2_ with CV cycles in H_2_^16^O-based 0.1 M KOH electrolyte. The expected ^18^O fractions are based on the natural isotope abundance and on the measured value from DEMS experiment of unlabelled LiNiO_2_ in the ^16^O-based electrolyte.

First-principles DFT calculations were employed to investigate the underlying mechanism of the enhanced OER activity of delithiated LiNiO_2_ with respect to the reaction steps. Similar to our previous work^11,62,63^, three scenarios were considered for the OER mechanisms involving four proton-electron transfer steps: metal-site adsorbate evolution (MAE) mechanism, lattice-oxygen-vacancy-site (LOV) mechanism, and metal-and lattice-oxygen-vacancy-site (MLOV) mechanism. In these mechanisms, the adsorbate sites of the reaction steps are different: only the Ni sites in MAE, oxygen sites in LOV, and both in MLOV (Fig. 7a). We built models for perfect LiNiO_2_ and delithiated Li_x_NiO_2_ to investigate the effects of the unsaturated Ni^IV^ state generated during OER. The (102) plane of LiNiO_2_ with a 50% lithium vacancy was used to model the surface reaction pathways on delithiated Li_x_NiO_2_ (x = 0.5, Supplementary Fig. 32), as the delithiation and oxidation of LiNiO_2_ and the concomitant OER all start from the edge^25,52^.

The Gibbs free energy differences (ΔG) of each OER step via the three OER mechanisms for Li_0.5_NiO_2_ and LiNiO_2_ are shown in Fig. 7b, respectively. First, regardless of the OER pathway, the potential-limiting step of Li_0.5_NiO_2_ possess lower overpotential than that of LiNiO_2_, in agreement with the operando measurement results indicating delithiated Li_x_NiO_2_ as the actual active phase for OER. Second, among the three OER scenarios for Li_0.5_NiO_2_ the MLOV route shows the lowest overpotential (0.69 V), suggesting that the OER via MLOV prevails on the delithiated Li_0.5_NiO_2_, and that the potential-limiting step is the adsorption of *OH (step 3). Third, for the OER on LiNiO_2_, the MLOV route also prevails, but the potential-limiting step switches to the formation of *OO intermediates and oxygen vacancies (step 1).

The transition of the potential-limiting step of LiNiO_2_ before and after delithiation can be attributed to the double ligand hole state induced by the presence of Ni^IV^ ions in Li_0.5_NiO_2_. In the electronic structure of octahedral-symmetric [NiO_6_] system, the charge-transfer energy from the O 2*p* to Ni 3*d* orbitals decreases with the increased valence state of the Ni ion^4,20,52^. The LiNiO_2_ system is already in the negative charge-transfer regime, and consequently, the Ni-O interaction is highly covalent^14,17^, in which Ni^III^ adopts an average configuration close to 3*d*^8^*L* (rather than the formal 3*d*^7^), leaving a ligand hole residing on the O 2*p* orbitals. The covalency increases further when the Ni^III^ is (partly) oxidised to Ni^IV^ via LiNiO_2_ delithiation, and the resulting Ni^IV^ was endowed with an average configuration of 3*d*^8^*L*^2^, that is, a double ligand hole state in O 2*p*. The high unoccupied density state with the O 2*p* ligand character in Li_x_NiO_2_ activates lattice oxygen, favouring direct coupling with oxygenated intermediates and the formation of oxygen vacancies (i.e. step 1 of the MLOV route), which are the potential-limiting step of LiNiO_2_. The prediction of oxygen vacancies formation during OER is in good agreement with the experimental observation of operando XAS (Fig. 6c). Thus, density-functional theory indicates that the double ligand holes created under OER condition easily activates lattice oxygen, accelerating the formation of O-O binding and oxygen vacancies, and the presence of double ligand hole states is closely correlated to the high OER activity of the delithiated Li_x_NiO_2_.

To confirm the participation of lattice oxygen during OER, we performed ^18^O-isotope labelling experiments using in situ differential electrochemical mass spectrometry (DEMS). The experiments were first carried out on unlabelled LiNiO_2_ in an ^18^O-enriched KOH electrolyte (prepared using 99% H_2_^18^O) over six consecutive CV cycles from 1.4 V to 1.8 V. Figure 7c shows the first CV and simultaneous mass spectrometric cyclic voltammogram (MSCV) in the time domain. In the MSCV curves, the mass signal m/z = 34 of ^16^O^18^O, which is mainly originated from the coupling of ^16^O of the LiNiO_2_ lattice and ^18^O of the electrolyte, shows significant ion current, with ^36^O_2_ as the dominant molecular oxygen isotope and ^32^O_2_ within the noise level. The measured ^16^O^18^O mass signal was also compared with the theoretically expected profile of the oxidation of the ^18^O-enriched electrolyte (1.18% ^16^O abundance, the inset of Fig. 7c), revealing a significant excess of the ^16^O^18^O ion charge. This evidences that the lattice oxygen plays a direct role in forming O_2_ during OER^4,10,64^. Furthermore, after the lattice ^16^O was partially replaced by ^18^O from the electrolyte, the resulting ^18^O-labelled LiNiO_2_ was cycled in a H_2_^16^O-based KOH electrolyte for another six consecutive cycles to study the reverse oxygen isotope replacement (Supplementary Fig. 33). The first-cycle data reveal a nearly two-fold ^18^O abundance than the expected ^18^O fractions from the natural isotope abundance and from the measured value of unlabelled LiNiO_2_ in the same O^16^-based electrolyte (Supplementary Fig. 34 and Supplementary Equation S28). The ^18^O isotope excess gradually decreases with the number of cycles Fig. 7d), indicating the consumption of ^18^O on the labelled LiNiO_2_ in forming O_2_. Therefore, the two-way isotope labelling experiments confirm the participation of LiNiO_2_ lattice oxygen in the OER, supporting the predicted MLOV mechanism and activated lattice-oxygen in the presence of double ligand holes.

### Fe^3+^ impurity effects

It is widely accepted that the incorporation of Fe^3+^ into Ni (oxy)hydroxides promote OER activity by forming synergetic active centres of oxygen-bridged Ni and Fe sites^56,65^, and the activity can be further enhanced via structural ordering, as recently proposed by Lee et al.^58^. Structurally intact Li_x_NiO_2_ with stabilised double ligand holes could be a better platform for forming such synergetic centres than the usually amorphous NiOOH.

**Fig. 8:**
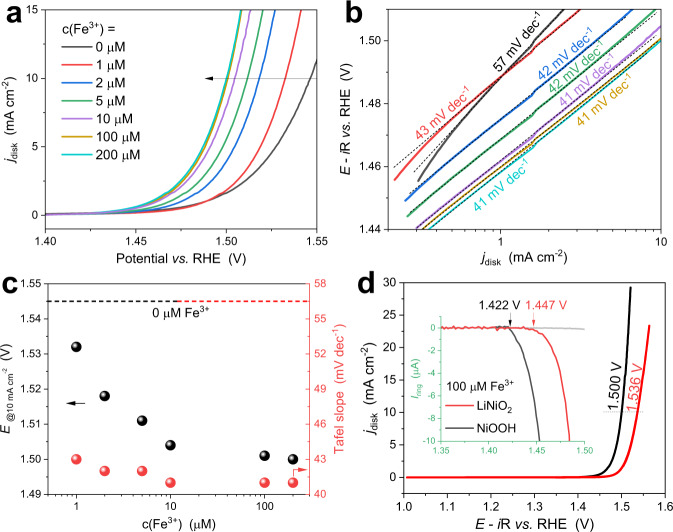
Promotional effects of Fe^3+^ on the OER activity of LiNiO_2_. **a** LSVs and **b** Tafel plots of LiNiO_2_ in Fe-free 1 M KOH with the addition of Fe^3+^ solutions (prepared from ultrapure water and Fe(NO_3_)_3_ ∙ 9H_2_O, 99.999%-Fe, PURATREM, Strem). **c** Potentials at 10 mA cm^−2^ and Tafel slopes as a function of Fe^3+^ concentrations. **d** Comparison of the Fe^3+^ promotional effects on LiNiO_2_ and NiOOH. Note that 100 µM Fe^3+^ reaches the saturated concentration of Fe(OH)_4_^-^ (the predominant Fe^3+^ species in alkaline solution)^70^.

Figure 8a compares the linear sweep voltammograms of LiNiO_2_ titrated with Fe^3+^ in purified 1 M KOH. Similar to the reported Ni (oxy)hydroxides, the OER activity of LiNiO_2_ is strongly correlated with the Fe^3+^ concentration in terms of E(@10 mA cm^−2^) and Tafel slopes (Fig. 8a–c): (1) E(@10 mA cm^−2^) decreases with the Fe^3+^ concentration and saturated at 1.500 V in the presence of 100 µM Fe^3+^ (45 mV lower than that in the Fe-free electrolyte, Figs. 8a), and (2) the Tafel slope plummets from 57 to 43 mV dec^−1^ in the presence of only 1 µM Fe^3+^, and largely remains constant up to a Fe^3+^ concentration of 200 µM (Fig. 8b). The decreased Tafel slope at such a low Fe^3+^ concentration indicates that OER is switched to a Fe-involving electrocatalytic site with higher activity, and the number of such new active sites increases with Fe^3+^ concentration, resulting in Fe-concentration-dependent E(@10 mA cm^−2^). For comparison, NiOOH was also measured in 1 M KOH containing 100 µM Fe^3+^. Although the overall activity is highly promoted as expected (Supplementary Fig. 35), NiOOH-Fe^3+^ shows 36 mV higher than LiNiO_2_-Fe^3+^ in E(@10 mA cm^−2^) and 25 mV in OER onset potential (Fig. 8d), proving LiNiO_2_ an excellent platform for forming Ni-Fe active sites. In addition, the presence of high-valent Ni in Ni-Fe active sites is also found to promote electrocatalytic durability^30^. The promotional mechanism and underlying role of double ligand holes in Ni-Fe lithiated oxide systems are also of interest and related studies are currently underway.

In conclusion, a comprehensive picture correlating electrochemical properties and operando spectroscopies is present to uncover the pristine activity, actual active species, and reaction centres of high-entropy LiNiO_2_ for OER. Pristine LiNiO_2_ with single-crystalline R-3m structure and with pure Ni^III^ was prepared and characterized unambiguously using electron microscopy, XRD, and XAS. The best and real OER activity of LiNiO_2_ is achieved when the electrochemical redox of Ni^2+^/Ni^III^ and the introduction of Fe impurities are prevented, and the activity is found to be comparable with that of the state-of-the-art IrO_2_ and much higher than that of the conventional NiOOH in terms of overpotentials, Tafel slopes and OER onset potentials.*,* Operando XRD, XAS, and Raman spectra were combined to probe the active species and reaction centre. The in situ delithiation, Ni^III^ oxidation and oxygen-vacancy formation in LiNiO_2_ under OER were found to coincide with OER for the first time, and the active species was then identified as delithiated yet structurally intact Li_x_NiO_2_ with high-valent Ni^IV^ and double ligand holes, which is also supported by the prevalence of the MLOV OER mechanism from DFT calculation and by the activated lattice oxygen from in situ DEMS measurements. This unique Li_x_NiO_2_ active sites were also found to be superior to those of NiOOH for the formation of a synergetic electrocatalyst with Fe^3+^. This study elucidates the electrocatalysis of lithiated metal oxides in OER and highlights the importance of considerate electrochemical measurements and complementary operando techniques in understanding the pristine electrochemistry and actual active species.

## Methods

### Preparation of LiNiO_2_

The pristine LiNiO_2_ was synthesized using a solid-state reaction method with two annealing steps. NiO and LiOH were grinded and mixed by a mortar and pestle for 30 min, and transferred into a tubular furnace. A 15% excess of the Li precursor was added to compensate for the evaporation of Li_2_O and/or Li_2_O_2_ during annealing. After the furnace was purged with O_2_ (100 sccm) for 15 min, under such O_2_ flow the temperature was increased to 750 °C with a ramping rate of 5 °C min^−1^ and held at this temperature for 72 h. The second annealing step was carried out at ~100 bar O_2_ pressure with cooling naturally to room temperature.

### Structural characterisation

Lab-based X-ray diffraction (XRD) patterns were obtained using a Bruker D8 Advance diffractometer with Cu K_α_ radiation (λ = 1.5418 Å) and a LYNXEYE detector, and diffraction peaks from Cu K_α2_ was stripped off using Jade 6 software. Transmission electron microscopy (TEM) measurements, including low-magnification TEM, high-resolution TEM (HRTEM) and selected area electron diffraction (SAED), were carried out on a FEI Tecnai G2 F20 S-TWIN electron microscope. Scanning electron microscopy (SEM) measurements were performed on a Zeiss Crossbeam 540. High-angle annular dark-field scanning transmission electron microscopy (HAADF-STEM) images were recorded using an aberration-corrected FEI Themis Z STEM. The microscope was operated with an accelerating voltage of 60 kV and the HAADF detector angle range was 50–200 mrad. Note that the accelerating voltage of STEM was selected as 60 kV to prevent beam damage, which is reported to induce Ni displacement and oxygen release on LiNiO_2_ after long exposure to electron irradiation^66^. X-ray absorption spectra (XAS) at the Ni *K* edge were measured in the transmission mode at TPS 44 A beamline of National Synchrotron Radiation Research Centre (NSRRC, Hsinchu), and using an in-house lab-based X-ray absorption spectrometer (Supplementary Fig. 36). The electron storage ring of the TPS was operated at 3.0 GeV with a ring current of ~500 mA. The measured spectra were processed and analysed using ATHENA and ARTEMIS (Demeter software package)^67^. Soft XAS (sXAS) at the Ni *L*_*3,2*_ edge and the O *K* edge were collected at TLS 11 A and 20 A beamlines of the NSRRC. The Ni *L* edge data of γ-NiOOH and KNiIO_6_ were measured in fluorescence mode and inverse partial fluorescence yield mode, respectively, to reduce the spectral distortion of surface Ni^2+^.

### Electrochemical measurements

Electrochemical measurements were performed on a standard three-electrode setup connected to a PGSTAT302N potentiostat (Metrohm Autolab, equipped with FRA32M and BA modules). A Pt gauze was used as the counter electrode, and a Hg|HgO electrode (in 1 M KOH) as the reference electrode. The potential measured using the Hg|HgO reference electrode, E(V vs. Hg|HgO, 1 M KOH), was converted into RHE scale in 1 M KOH, E(V vs. RHE), using the following equation,

E(V vs. RHE) = E(V vs. Hg|HgO, 1 M KOH) + 0.0977 V + 0.05916 pH (V) **(1)**, where the pH of the purified 1 M KOH is measured as 13.73 at 25 °C. The values of potential in the text are thus referenced against RHE, unless stated otherwise. The contact and solution resistance were determined using electrochemical impedance spectroscopy under open circuit potential (OCP) before each voltammetry measurement, and the voltammograms were corrected by IR drops.

The working electrode was prepared by drop-casting 10 μL of a catalyst ink onto a polished rotating ring-disk electrode (RRDE, with a glassy carbon disk and a Pt ring) and drying under ambient condition to obtain an even film. The catalyst ink was prepared by dispersing 5 mg of each sample and 5 mg carbon (Vulcan XC-72R) into 1 mL isopropanol alcohol (IPA) aqueous solution (25 vol% of IPA) containing 40 μL 5 wt% Nafion solution.

Linear sweep voltammograms and cyclic voltammograms (CV) were collected using a RRDE configuration with 5 mV s^−1^ in Ar-saturated 1 M purified KOH solution at 25 °C. The voltammograms were collected at 1600 rpm to promptly remove the generated O_2_, and the disk potential was swept from the corresponding OCP of each sample, while the ring potential was held at −0.5 V (vs. Hg|HgO, 1 M KOH) to electrochemically reduce and detect the O_2_ evolved from the disk. For the long-term stability test, the working electrode was prepared using carbon paper (Toray TGP-H-060) with the same catalyst loading and ink recipe as the RRDE, and the stability was evaluated using chronopotentiometry at 10 mA cm^−2^ for 48 h, before and after which LSV curves were measured with 5 mV s^−1^. The OER activity of NiOOH and IrO_2_ (99.9%, Adamas-beta) was measured for comparison. The NiOOH was prepared by potential cycling NiO nanopowder (50 nm, 99.9% metal basis, Aladdin) in 1 M KOH between 1.01 V and 1.66 V for at least 40 cycles until the Ni^2+^/Ni^III^ redox couple at ~1.32 V was fully developed (Supplementary Fig. 37).

To avoid the inference of Fe impurities on the measured OER activity, the electrochemical cell was cleaned by concentrated H_2_SO_4_ overnight and boiled with ultrapure H_2_O (18.2 MΩ cm) for at least 3 times to remove residual H_2_SO_4_ and other impurities, and the KOH solution was purified using Trotochaud’s method^38^. Briefly, 1 M KOH solution was first prepared from semiconductor grade KOH (99.9996% metal basis, 4 ppm total metal impurity excluding Na^+^, Aladdin) and ultrapure water. Into a H_2_SO_4_-rinsed 50 mL polypropylene centrifuge tube, ~2 g of Ni(NO_3_)_2_·6H_2_O (99.9985%, PURATREM, STREM) were dissolved in ~4 mL ultrapure H_2_O and mixed with 20 mL 1 M KOH. The resulting Ni(OH)_2_ suspension was shaken and centrifuged, and the supernatant was decanted. The Ni(OH)_2_ was then washed 3 times with ~20 mL ultrapure H_2_O and ~2 mL 1 M KOH, and transferred to a H_2_SO_4_-rinsed 100 mL polypropylene bottle. The bottle was filled with 75 mL 1 M KOH for purification, and the freshly prepared and washed Ni(OH)_2_ was redispersed and shaken for 10 min, followed by resting overnight.

### Operando and online characterisation

Operando synchrotron XRD patterns were collected at TLS 01C2 beamlines of the NSRRC. The XRD experiments were performed at an incident beam energy of 18 keV using a mar345 image plate area detector. The diffraction intensity and the peak shape were calibrated with a CeO_2_ reference sample (SRM 674b). Operando Quick*-*XAS measurements at Ni K were carried out in the transmission mode at TPS 44 A beamline of the NSRRC, and 120 XAS spectra collected within 1 min were merged to achieve high data quality. Operando Raman spectra were collected using a Horiba LabRAM HR Evolution confocal Raman microscope with an excitation wavelength of 473 nm. The spectra resolution is ~1 cm^−1^, and the spectral shifts were calibrated routinely using silicon wafer (520.7 cm^−1^). The operando Raman experiments were performed in a homemade spectro-electrochemistry cell, consisting of a Au disk working electrode (5 mm), a Pt wire counter electrode and a Ag|AgCl (saturated KCl) reference electrode. Online differential electrochemical mass spectrometry (DEMS) measurements were carried out using a QAS-100 equipped with a PrismaPro quadrupole mass spectrometer (Linglu Instruments). The O^18^-labeled LiNiO_2_ was prepared by cycling the unlabelled one to the OER range (e.g., 1.8 V) in ^18^O-enriched electrolyte, and before the experiment in the ^16^O-based electrolyte, rinsing and drying steps were conducted to remove free ^18^O species.

### Computation details

The present calculations employ the Vienna ab initio Simulation Package (VASP) implementation of density functional theory (DFT) in conjunction with the projector augmented wave (PAW) formalism^68^. The H 1*s*^1^, Li 2*s*^1^, O 2*s*^2^2*p*^4^, and Ni 4*s*^2^3*d*^8^ states are treated as valence electrons. The electronic wave functions were expanded in plane waves using an energy cutoff of 520 eV, and the force and energy convergence criteria were set to 0.02 eV Å^−1^ and 10^−5^ eV respectively. To overcome the self-interaction error of the exchange correlation functional, we employed the Hubbard *U* model for describing the strong correlation of the localized Ni 3*d* states, and set the value of *U*_*eff*_ = (*U* − J) to 6.40 eV according to previous work^46^. The reciprocal space was sampled using a 4×4×1 Monkhorst–Pack *k*-point mesh for DFT + U calculations. To simulate the valence states of Ni in real catalytic environment, we built the Li_0.5_NiO_2_ system containing 50% lithium vacancy. The periodic slab models of LiNiO_2_ and Li_0.5_NiO_2_ system with 9 Ni sites per surface, with two layers at the bottom fixed during the relaxation. To prevent spurious interactions, the thickness of vacuum spacing was ~15 Å in the *z-direction*. We calculated the Gibbs free-energy differences (∆G) using the computational hydrogen electrode model under standard conditions^69^, with the voltage applied U_RHE_ = 0 and 1.23 V. The calculation details regarding ∆G can be found in the Supplementary Information.

## Supplementary information

Supplemenetary information

## Data Availability

The data that support the findings of this study are available either from the Supplementary Information and the Source data or from the corresponding author upon reasonable request. Source data are provided with this paper.

## Source data

Source data
