# Supplementary material for: Unusual double ligand holes as catalytic active sites in LiNiO2
Source: Nat Commun. 2023 Apr 13;14:2112. doi: 10.1038/s41467-023-37775-4 (PMC10102180; doi:10.1038/s41467-023-37775-4)
Supplement: Supplementary file 1 — Supplemenetary information [file 41467_2023_37775_MOESM1_ESM.pdf]

Supplementary Information

Unusual double ligand holes as catalytic active sites in $LiNiO_2$

Haoliang Huang[1‡], Yu-Chung Chang[2‡], Yu-Cheng Huang[3], Lili Li[1], Alexander C. Komarek[4], Liu Hao Tjeng[4], Yuki Orikasa[5], Chih-Wen Pao[2], Ting-Shan Chan[2], Jin-Ming Chen[2], Shu-Chih Haw[2], Jing Zhou[1], Yifeng Wang[1], Hong-Ji Lin[2], Chien-Te Chen[2], Chung-Li Dong[3], Chang-Yang Kuo[2,6], Jian-Qiang Wang[1,7], Zhiwei Hu[4], Linjuan Zhang*[1,7]

[1]Key Laboratory of Interfacial Physics and Technology, Shanghai Institute of Applied Physics, Chinese Academy of Sciences, Shanghai 201800, China

[2]National Synchrotron Radiation Research Center, Hsinchu, Taiwan 30076, R. O. C.

[3]Department of Physics, Tamkang University, New Taipei City, Taiwan 25137, R. O. C.

[4]Max Planck Institute for Chemical Physics of Solids, Dresden 01187, Germany

[5]Department of Applied Chemistry, Ritsumeikan University, Kusatsu, Shiga 535-8577, Japan

[6]Department of Electrophysics, National Yang Ming Chiao Tung University, Hsinchu, Taiwan 30010, R. O. C.

[7]University of Chinese Academy of Sciences, Beijing 10049, China

‡H.H. and Y.-C. C. contributed equally

*Linjuan Zhang: email: zhanglinjuan@sinap.ac.cn

Supplementary Note 1: Free energy calculations

The elementary steps of metal-site adsorbate evolution mechanism (MAE) mechanisms were listed below:

$$H_2O + * \rightarrow OH* + e^- + H^+ \quad (S1)$$

$$OH* \rightarrow O* + e^- + H^+ \quad (S2)$$

$$H_2O + O* \rightarrow OOH* + e^- + H^+ \quad (S3)$$

$$OOH* \rightarrow O_2 + e^- + H^+ \quad (S4)$$

The OH*, O* and OOH* represent OH, O and OOH species adsorbed on the metal-site (*) on the surface, respectively.

The Gibbs free energy changes (ΔG) were calculated by the following Equations:

$$\Delta G_1 = \Delta G_{[OH*]} - eU \quad (S5)$$

$$\Delta G_2 = \Delta G_{[O*]} - \Delta G_{[OH*]} - eU \quad (S6)$$

$$\Delta G_3 = \Delta G[_{OOH*]} - \Delta G_{[O*]} - eU \quad (S7)$$

$$\Delta G_4 = -2\,\Delta g_{H_2O}^{exp} - \Delta G[_{OOH*]} - eU \quad (S8)$$

where U is the potential measured against RHE at standard condition (T = 298.15 K, P = 1 bar, pH = 0), $\Delta g_{H_2O}^{exp}$ is the experimental Gibbs free energy of formation of water molecules. The ΔG of these intermediates include zero-point energy (ZPE) and entropy corrections according to $\Delta G_i = \Delta E_i + \Delta ZPE_i - T\Delta S_i$, where the energy differences ΔE_i are calculated with respect to H_2O and H_2 (at U = 0 and pH = 0). The theoretical overpotential is defined as the lowest potential at which all reaction steps are thermodynamically downhill.

The lattice-oxygen-vacancy site mechanism (LOV) mechanisms were listed below:

$$OH^{VO} \rightarrow O^{VO} + e^- + H^+ \quad (S9)$$

$$H_2O + O^{VO} \rightarrow OOH^{VO} + e^- + H^+ \quad (S10)$$

$$OOH^{VO} \rightarrow V_O + O_2 + e^- + H^+ \quad (S11)$$

$$V_O + H_2O \rightarrow OH^{VO} + e^- + H^+ \quad (S12)$$

Here, OH^{VO}, O^{VO} and OOH^{VO} represent OH, O and OOH species adsorbed locate at the lattice oxygen vacancy (Ov) on the surface, respectively. The oxygen vacancy is easy to fill by OH in alkaline environment and start the OER reaction from the situation of OH^{VO} .

The Gibbs free energy changes (ΔG) were calculated by the following Equations:

$$\Delta G_1 = \Delta G_{[O^{VO}]} - \Delta G_{[OH^{VO}]} - eU \quad (S13)$$

$$\Delta G_2 = \Delta G_{[OOH^{VO}]} - \Delta G_{[O^{VO}]} - eU \quad (S14)$$

$$\Delta G_3 = -2\,\Delta g_{H_2O}^{exp} - \Delta G_{[OOH^{VO}]} - eU \quad (S15)$$

$$\Delta G_4 = \Delta G_{[OH^{VO}]} - eU \quad (S16)$$

The metal-and-lattice-oxygen-vacancy-site (MLOV) mechanisms were listed below:

$$OH^* \rightarrow [OO^* + V_O] + e^- + H^+ \quad (S17)$$

$$H_2O + [OO^* + V_O] \rightarrow O_2 + [* + OH^{VO}] + e^- + H^+ \quad (S18)$$

$$H_2O + [* + OH^{VO}] \rightarrow [OH^* + OH^{VO}] + e^- + H^+ \quad (S19)$$

$$[OH^* + OH^{VO}] \rightarrow OH^* + e^- + H^+ \quad (S20)$$

The Gibbs free energy changes (ΔG) were calculated by the following Equations:

$$\Delta G_1 = \Delta G_{[OO^*+Ov]} - \Delta G_{[OH^*]} - eU \quad (S21)$$

$$\Delta G_2 = -2\,\Delta g_{H_2O}^{exp} + \Delta G_{[*+OH^{VO}]} - \Delta G_{[OO^*+VO]} - eU \quad (S22)$$

$$\Delta G_3 = \Delta G[_{OH^*+OH^{VO}}] - \Delta G_{[*+OH^{VO}]} - eU \quad (S23)$$

$$\Delta G_4 = \Delta G_{[OH^*]} - \Delta G_{[OH^*+OH^{VO}]} - eU \quad (S24)$$

Supplementary Note 2: Calculation of atomic O isotope abundance and DEMS-derived O_2 isotopologue charges

The mass ion current of $^{16}O_2$, $^{16}O^{18}O$ and $^{18}O_2$ was recorded in the DEMS experiments, and the ^{18}O abundance in the $H_2{}^{18}O$ (99%) was certified by the manufacturer (Adamas-beta). The ^{16}O fraction from the $H_2{}^{18}O$ is then ~1%, and that from KOH (0.1 M) is 0.18%. So, the total ^{16}O percentage from the O^{18}-enriched 0.1 M KOH results in 1.18%.

In ^{18}O-enriched electrolyte where the $^{32}O_2$ signal is not detectable, the relationship between the atomic abundance of ^{16}O, ^{16}a, and the integrated DEMS charge, Q_{MS}, is

$$^{16}a = \frac{Q_{MS}(^{16}O^{18}O)}{2[Q_{MS}(^{16}O^{18}O) + Q_{MS}(^{18}O^{18}O)]} \tag{S25}$$

So, the theoretically expected $Q_{MS}(^{16}O^{18}O)$ and $i_{MS}(^{16}O^{18}O)$ purely from the oxidation of ^{18}O-enriched electrolyte are

$$Q_{MS}(^{16}O^{18}O) = \frac{2\,^{16}a}{1-2\,^{16}a}\,Q_{MS}(^{18}O^{18}O) \tag{S26}$$

$$i_{MS}(^{16}O^{18}O) = \frac{2\,^{16}a}{1-2\,^{16}a}\,i_{MS}(^{18}O^{18}O) = 2.42\%\,i_{MS}(^{18}O^{18}O) \tag{S27}$$

Similarly, the atomic abundance of ^{18}O, ^{18}a, in the ^{16}O-based electrolyte follows from the integrated DEMS charge as,

$$^{18}a = \frac{Q_{MS}(^{16}O^{18}O)}{2[Q_{MS}(^{16}O^{18}O) + Q_{MS}(^{16}O^{16}O)]} \tag{S28}$$

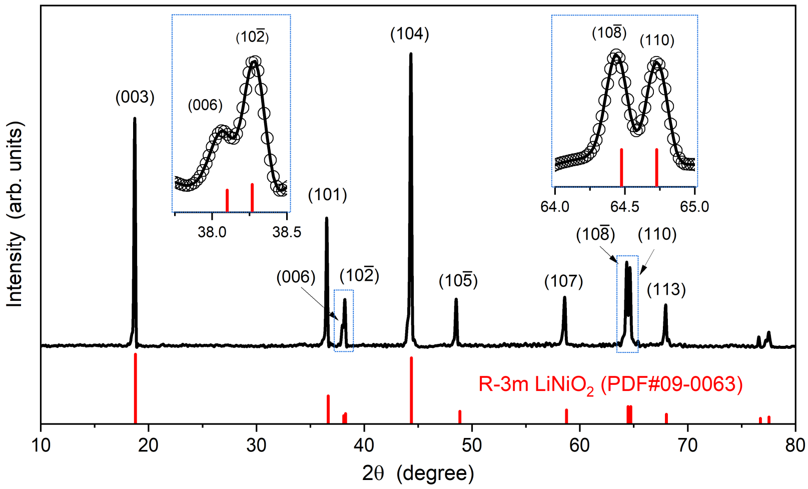

Supplementary Figure 1. A lab-based XRD pattern of $LiNiO_2$, along with the standard pattern of layered $LiNiO_2$ (R-3m, PDF#09-0063). Two regions at around 38.2° and around 64.6° are magnified in the inset. The well separation of (006) and (10-2) and of (10-8) and (110) indicates the layered R-3m structure with a low degree of Li/Ni intermixing. Diffraction peaks from Cu $K_{\alpha 2}$ radiation were stripped using Jade 6 software.

Supplementary Table 1. Refined structural parameters of the as-prepared $LiNiO_2$.

Atom	x	y	z	Occupancy	U_{iso}
**Li(1)**	0	0	0.00000	0.961	0.00717
**Ni(1)**	0	0	0.50000	1.003	0.00981
**O(1)**	0	0	0.24376	0.996	0.01339
**Ni(2)**	0	0	0.00000	0.039	0.01034

Refined lattice parameters: R-3m space group, a = b = 2.88336(7) Å, c = 14.2090(2) Å, α = β = 90°, γ = 120° and the unit cell volume V = 102.304(4) $Å^3$. R_w = 2.78% and GOF = 0.19.

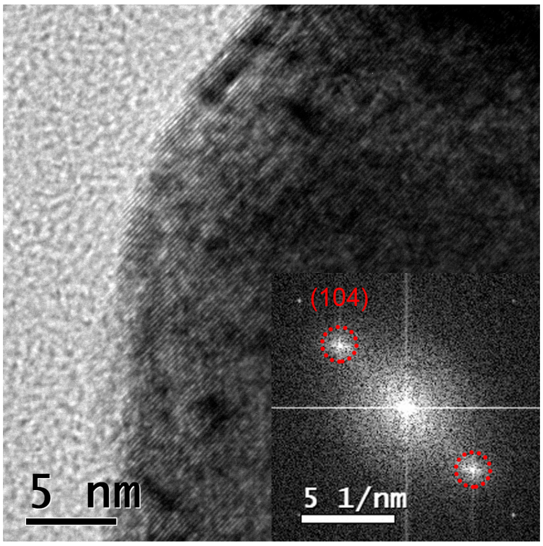

Supplementary Figure 2. A HRTEM image of $LiNiO_2$ showing the (104) lattice fringes, and the corresponding FFT pattern.

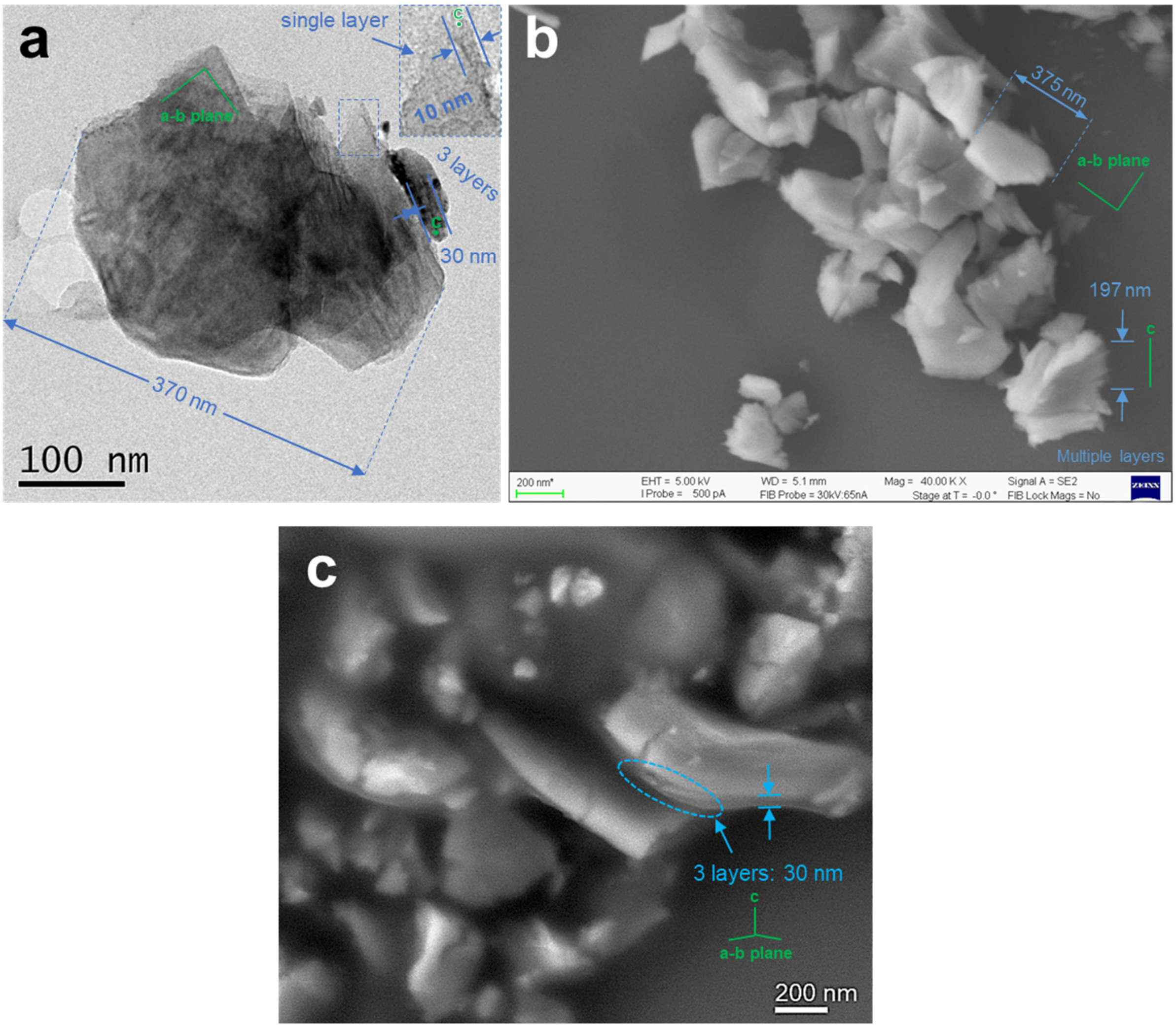

Supplementary Figure 3. (**a**) TEM and (**b**, **c**) SEM image of $LiNiO_2$.

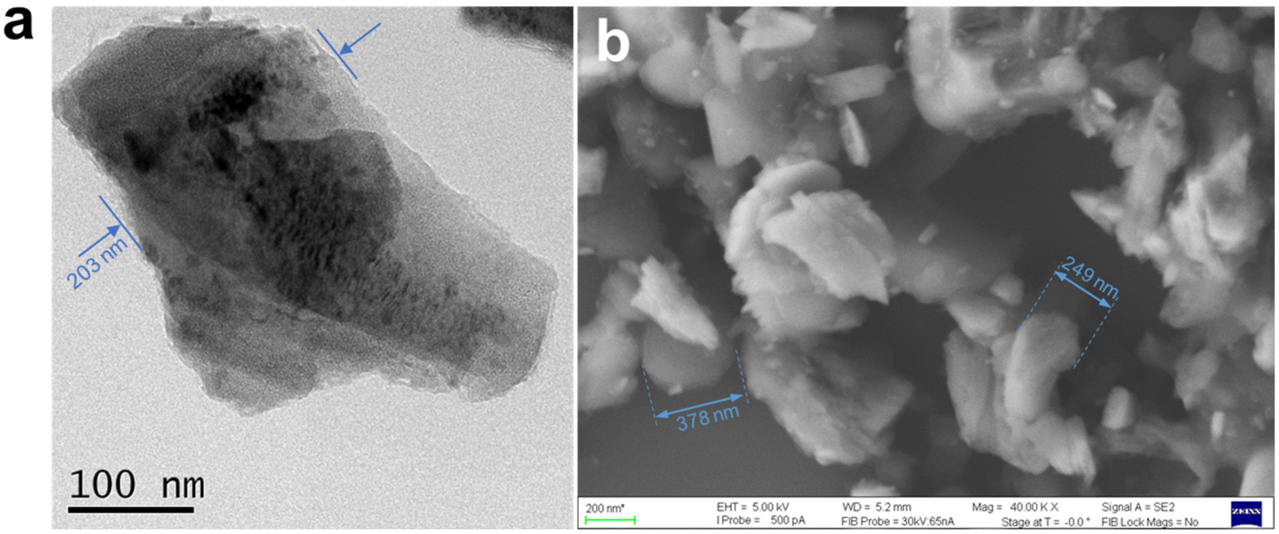

Supplementary Figure 4. **(a)** TEM and **(b)** SEM images of $LiNiO_2$-raw.

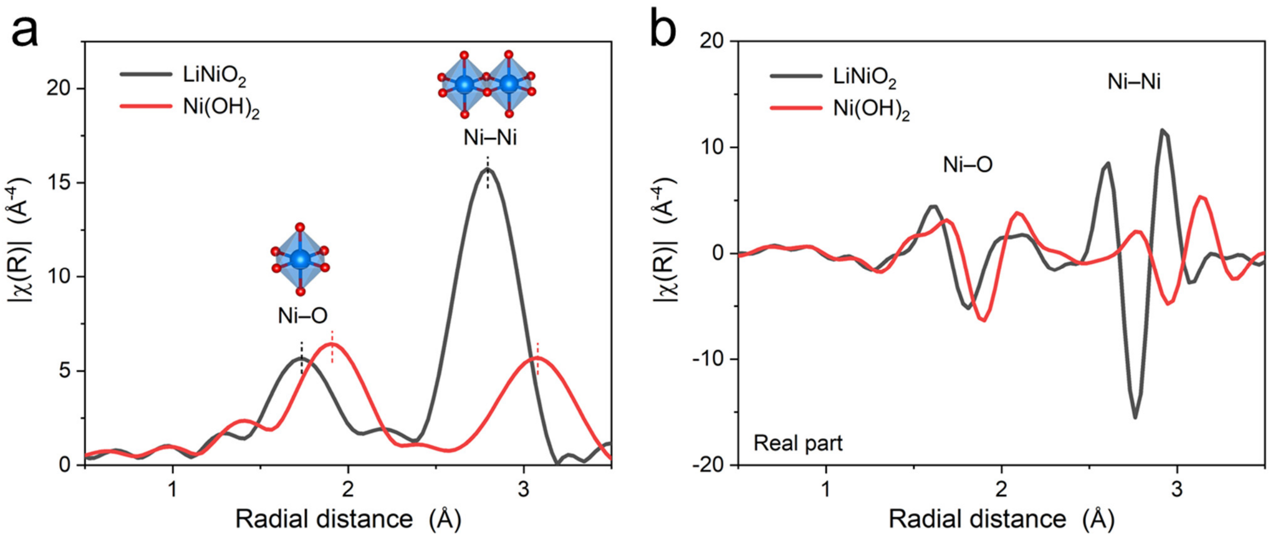

Supplementary Figure 5. Fourier transforms of k^3-weigted EXAFS spectra of $LiNiO_2$ and $Ni(OH)_2$, plotted as **(a)** the amplitude and **(b)** the real part.

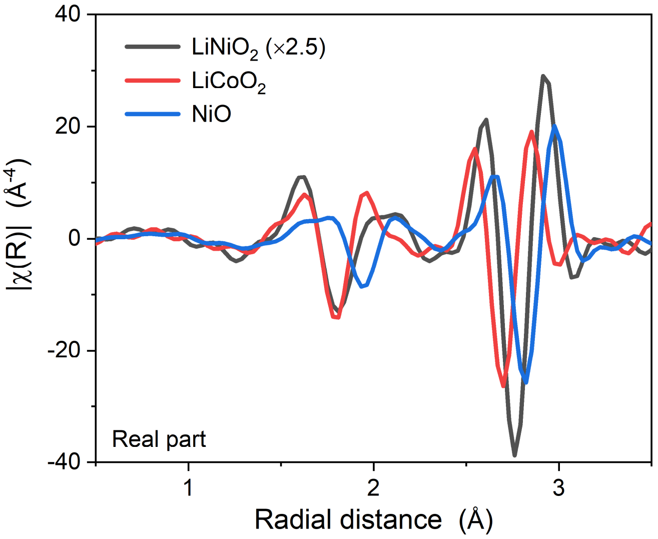

Supplementary Figure 6. Same as **Figure 3a**, but plotted as the real part of Fourier transforms.

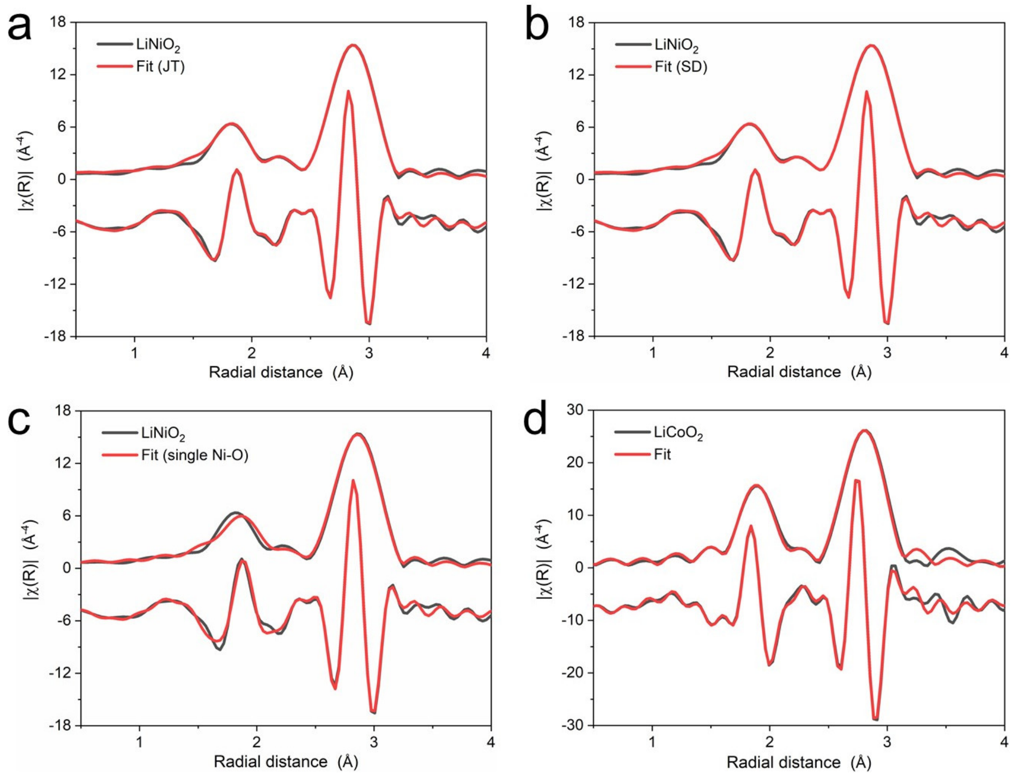

Supplementary Figure 7. Fits of *ex situ* EXAFS spectra of $LiNiO_2$ using different models of Ni-O bond distortion (**a**: Jahn-Teller effect, **b**: a size-disproportionated NiO_6 mode, and **c**: considering Ni-O distortion using the σ^2 of Ni−O) and (**d**) $LiCoO_2$. The fits are shown as the amplitude and the real part of R space. The Fourier transformation was k^3-weighted and performed over a k range of 3.5–14.0 Å^{-1} for $LiNiO_2$ and 3.7–14.6 Å^{-1} for $LiCoO_2$, and the fitting was carried out over a R range of 1–3 Å. The obtained structural parameters are listed in **Table 1**, and the real part plots of the Fourier transform is shifted downwards for clarity. The plots are phase-corrected using the corresponding metal–metal scattering path.

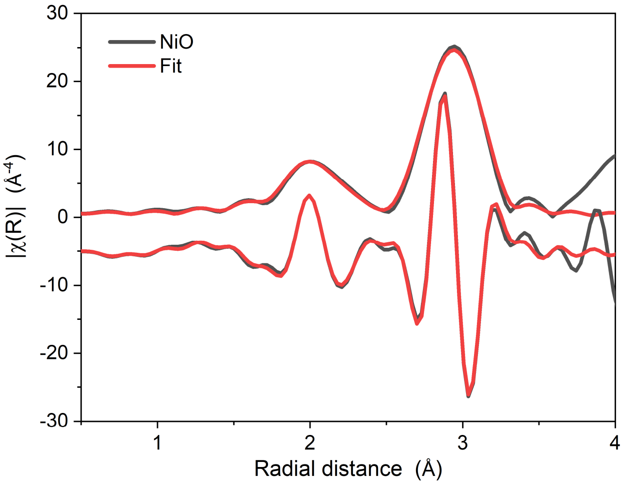

Supplementary Figure 8. Fits of EXAFS spectra of NiO. The fits are shown as the magnitude and the real part of R space. The Fourier transformation of NiO was k^3-weighted and performed over a k range of 3.4–14.0 Å^{-1} with phase correction using the Ni-Ni scattering path, and the fitting was carried out over a R range of 1–3 Å. The obtained structural parameters are listed in **Table 1**, and the real part plots of the Fourier transform is shifted downwards for clarity.

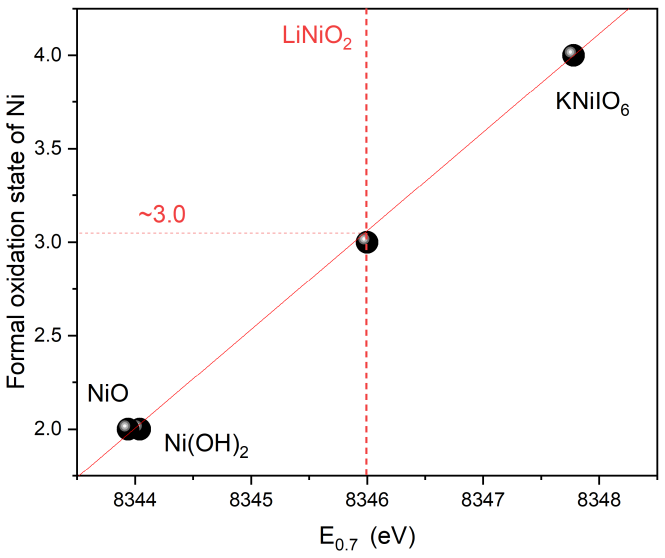

Supplementary Figure 9. Formal oxidation states of Ni as a function of edge energy at 0.7 jump height of ($E_{0.7}$), obtained from normalised XANES spectra at the Ni K edge.

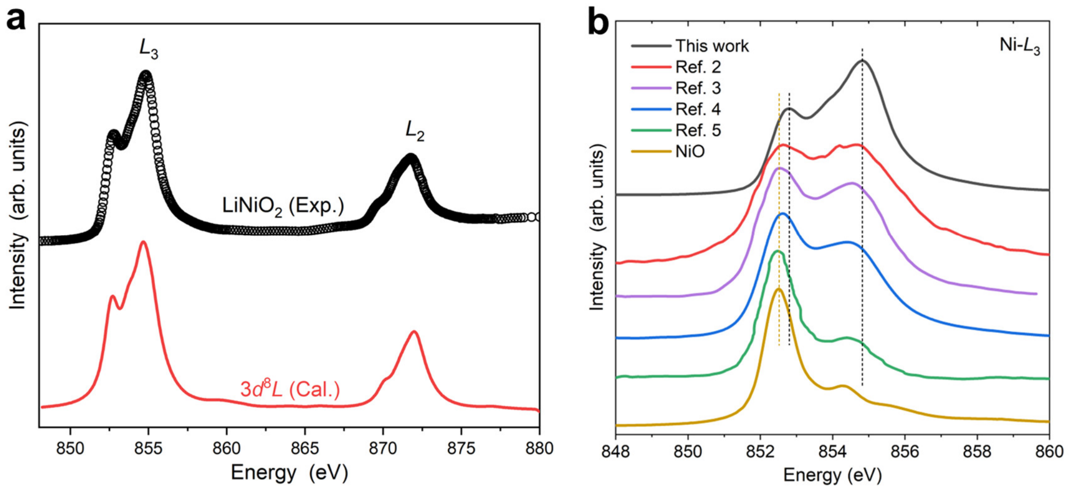

Supplementary Figure 10. (a) Total-electron-yield Ni $L_{3,2}$ sXAS of $LiNiO_2$, along with a simulated spectrum of nominal $3d^8L$ configurations using the full multiplet cluster calculation[1], which includes full intra-atomic multiplet interaction, crystal field interaction and covalence. (b) Comparison of Ni-L_3 sXAS of $LiNiO_2$ samples from this work and from previous work[2, 3, 4, 5].

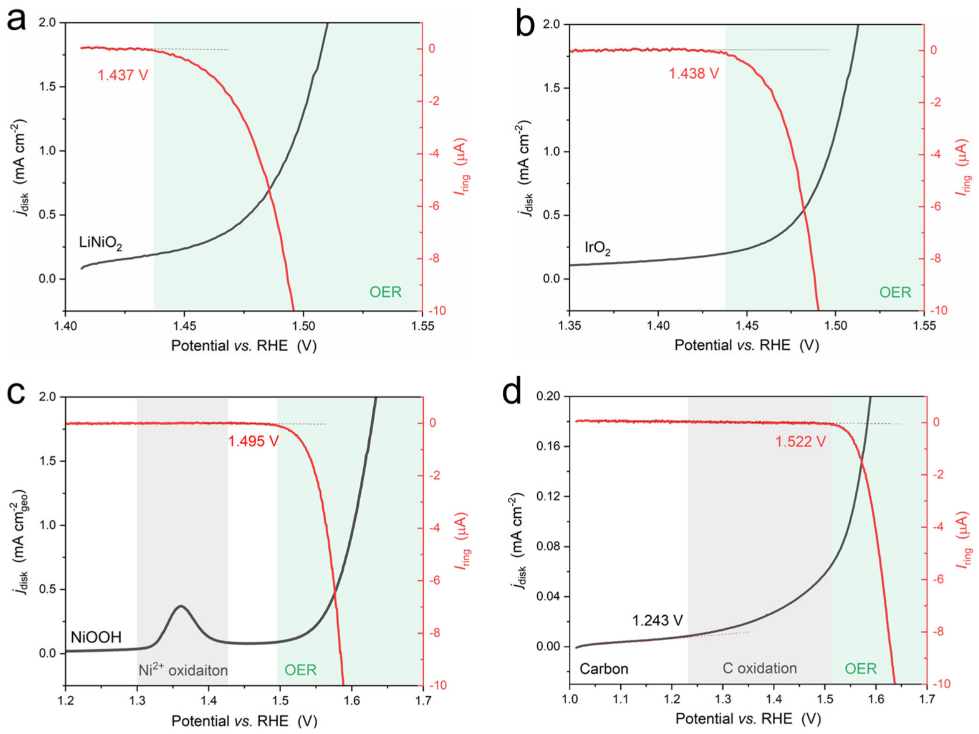

Supplementary Figure 11. RRDE linear sweep voltammograms of (**a**) $LiNiO_2$, (**b**) IrO_2, (**c**) NiO-derived NiOOH, and (**d**) Vulcan XC-72R carbon (0.25 mg cm^{-2} loading) in Ar-saturated 1 M KOH solution, with the disk current density shown in the left axis and the Pt ring current in the right axis. The onsets of the ORR current on the Pt ring were indicated, which were determined by extrapolating the baseline at low potentials with the consideration of the noise level of the voltammograms. The voltammograms were collected under 1600 rpm with a sweep rate of 5 mV s^{-1}. The oxide loading is ~0.25 mg cm^{-2}, with ~0.25 mg cm^{-2} carbon.

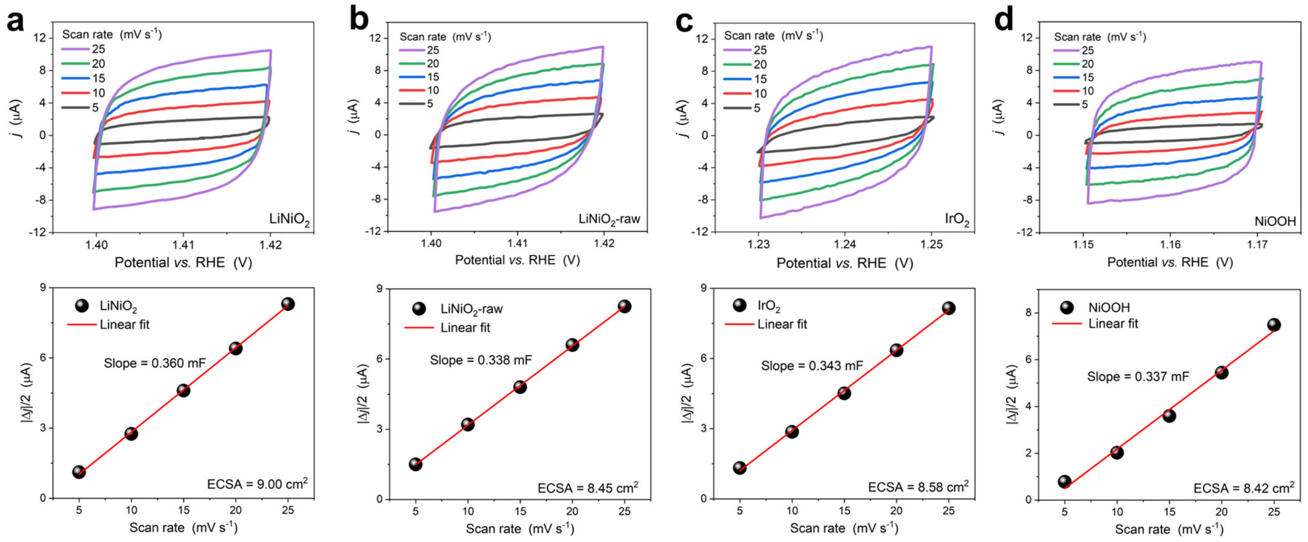

Supplementary Figure 12. CV curves of (a) $LiNiO_2$, (b) $LiNiO_2$-raw, (c) IrO_2 and (d) NiOOH with different scan rates, and the corresponding $|\Delta j|/2$ plotted against scan rates. The corresponding electrochemical surface areas (ECSA) were calculated using the following equation, ECSA = C_{dl}/C_s, where C_{dl} is the double layer capacitance, the slope of $|\Delta j|/2$~scan rate plot, and C_s is a reported constant of 0.04 mF cm^{-2}.

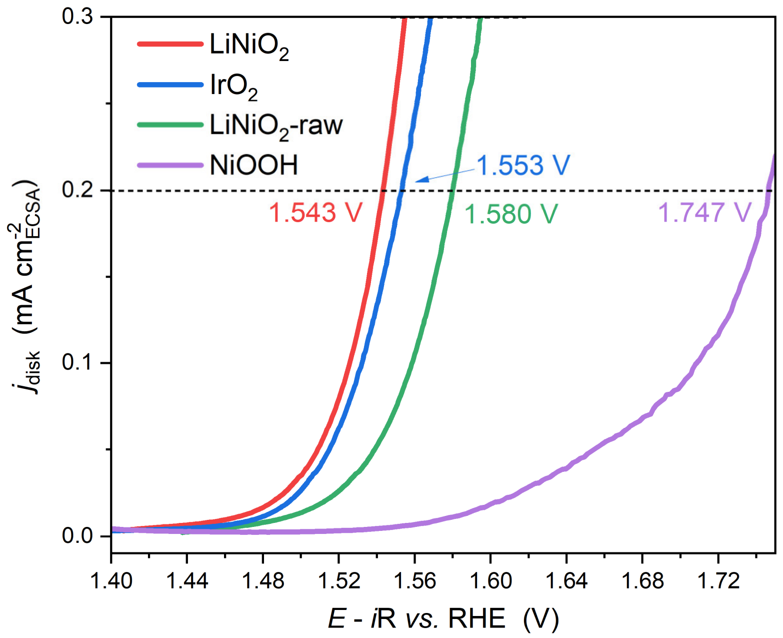

Supplementary Figure 13. ECSA-normalised LSV of $LiNiO_2$, $LiNiO_2$-raw, IrO_2 and NiOOH.

Supplementary Table 2. Comparison of reported $LiNiO_2$ and Fe-doped $LiNiO_2$ with the as-prepared $LiNiO_2$ in terms of E@10 mA cm^{-2} and Tafel slopes.

**Samples**	**KOH concentration**	***E* @ 10 mA cm^{-2} (V)**	**Tafel slope ($mV\ dec^{-1}$)**	**Reference**
$LiNiO_2$	1 M	1.545	57	This work
	0.1 M	1.568	60	
$LiNiO_2$-raw	1 M	1.585	66	
$LiNiO_2$	0.1 M [a]	1.657	82	Ref 6
$LiNi_{0.9}Fe_{0.1}O_2$		1.584	63	
$LiNi_{0.8}Fe_{0.2}O_2$		1.560	59	
$LiNi_{0.7}Fe_{0.3}O_2$		1.673	86	
LNO (Initial)	1 M [a]	1.580 (Initial)	63.4	Ref 7
LNO (500 cycles)		1.530 (500th cycles) [b]	47.2 [b]	
$LiNiO_2$	1 M [a]	1.60	56.6	Ref 4
$LiNi_{0.9}Fe_{0.1}O_2$		1.57	46.2	
$LiNiO_2$	0.1 M [a]	>1.7 V [c]	64.3	Ref 2
Layered $LiNiO_2$	0.1 M [a]	>1.8 V [c]	88	Ref 8
Disordered $LiNiO_2$		~1.69 (2nd cycles) [c], 1.63 (50th cycle)	63	
Pseudo-spinel $LiNiO_2$		1.57	62	

[a] unpurified KOH;

[b] suspected of accumulating Fe impurities with CV cycles

[c] estimated values

Supplementary Table 3. OER activity comparison of the as-prepared $LiNiO_2$ to $LiMO_2$ (M = Co and Fe), and *R*MO_3 (*R* = Rare earth elements, M = Fe, Co and Ni) and other unary 3*d* transition metal catalysts.

Samples	KOH concentration	*E* vs. RHE (V)	*j* (mA cm^{-2})	Reference
$LiNiO_2$	1 M	1.545	10	This work
	0.1 M	1.568	10	
$Ni(OH)_2$	1 M	~1.759	10	Ref 9
β-$Co(OH)_2$	0.1 M	~1.700	4.5	Ref 10
$Co(OH)_2$	1 M	~1.62	2.5	Ref 11
NiO_xH_y	1 M	~1.63	2.5	Ref 12
CoO_xH_y		~1.61	2	
FeO_xH_y		~1.60	2.5	
MnO_xH_y		~1.73	2.5	
$SrCoO_{3-\delta}$	0.1 M	~1.65	5.5	Ref 13
$LaCoO_3$	1 M	1.7 V	2	Ref 14
$LiCoO_2$	0.1 M [a]	1.660	10	Ref 15
$Li_2Co_2O_4$	1 M [a]	~1.59	10	Ref 16
LT-$LiCoO_2$	0.1 M [a]	~1.64	10	Ref 17
HT-$LiCoO_2$		~1.68		
Co_3O_4		~1.66		
$LiFeO_2$	0.1 M [a]	~1.60	0.09	Ref 18
$LaNiO_3$ film	0.1 M	~1.74	1	Ref 19
$PrNiO_3$ film		~1.72	1	
$NdNiO_3$ film		~1.73	1	
$LiMnO_2$	0.1 M [a]	~1.7	0.1	Ref 20
$LaMnO_3$	0.1 M [a]	~1.65	0.04	Ref 21
$Na_2Mn_3O_{7-\delta}$	1 M [a]	1.55	0.07	Ref 22
$Na_{1.5}Mn_3O_{7-\delta}$		1.55	1.30	
$NaMn_3O_{7-\delta}$		1.55	3.42	
$Na_{0.7}Mn_3O_{7-\delta}$		1.55	0.36	

[a] may contain Fe impurity

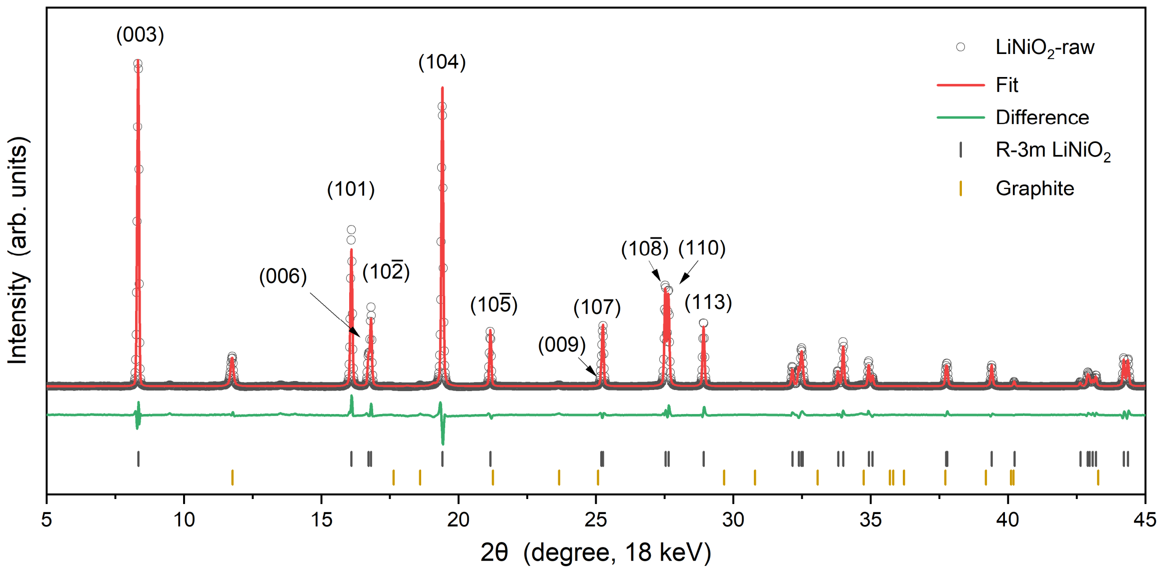

Supplementary Figure 14. A synchrotron XRD pattern of $LiNiO_2$-raw (18 keV incident beam energy, $\lambda = \sim 0.6888$ Å) and a Rietveld refinement fit with the refined structural parameters listed in **Supplementary Table 4**.

Supplementary Table 4. Refined structural parameters of the $LiNiO_2$-raw.

Atom	x	y	z	Occupancy
**Li(1)**	0	0	0.00000	0.948
**Ni(1)**	0	0	0.50000	0.9997
**O(1)**	0	0	0.24184	**0.954**
**Ni(2)**	0	0	0.00000	0.052

Refined lattice parameters: R-3m space group, a = b = 2.88358(6) Å, c = 14.2134(2) Å, $\alpha = \beta = 90°$, $\gamma = 120°$ and the unit cell volume V = 102.351(3) Å^3. R_w = 2.96% and GOF = 0.68.

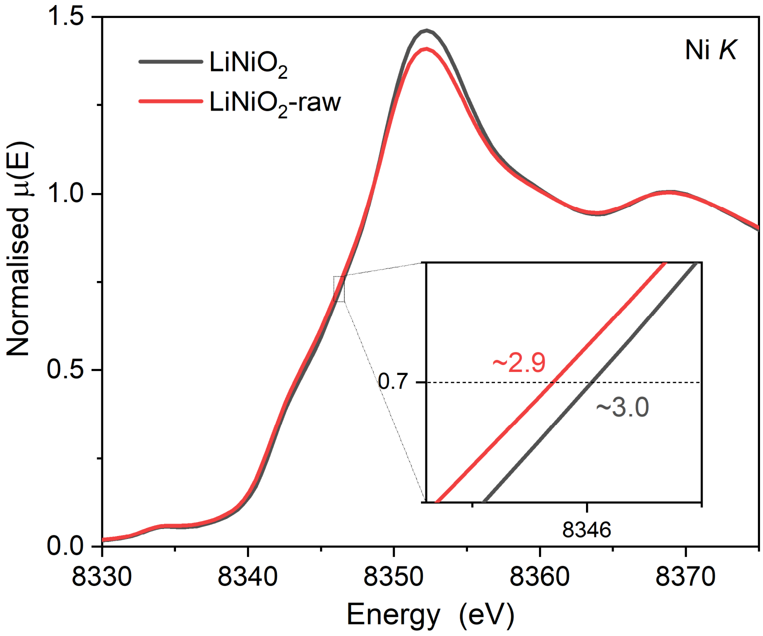

Supplementary Figure 15. XANES spectra of $LiNiO_2$ and $LiNiO_2$-raw. The latter shows lower edge relative to the former.

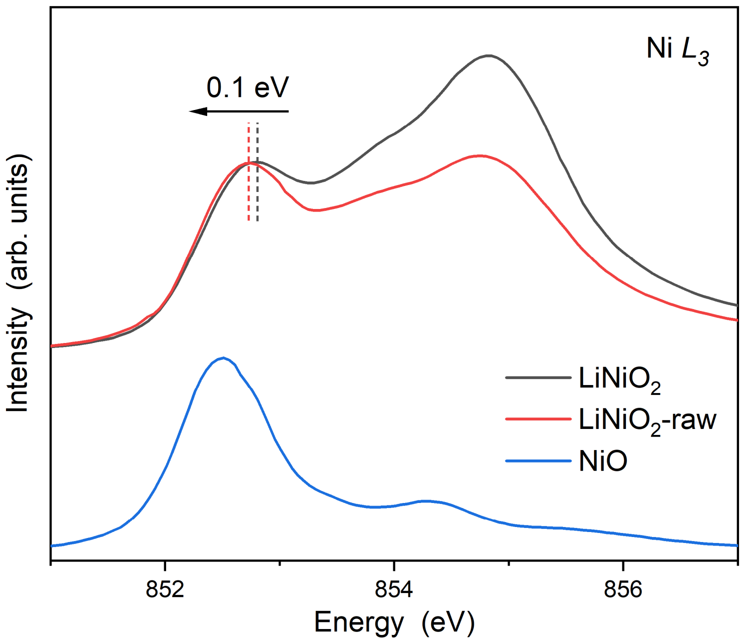

Supplementary Figure 16. TEY-sXAS of $LiNiO_2$ and $LiNiO_2$-raw at the Ni L_3 edge. The latter presents a large spectral weight and a lower energy shift of the lower energy peak, relative to the former.

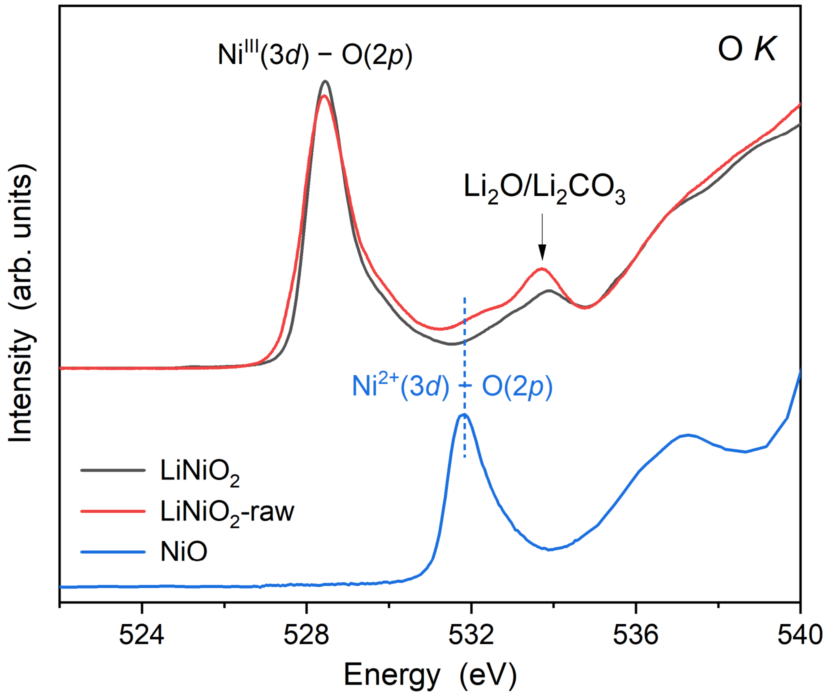

Supplementary Figure 17. TEY-sXAS of $LiNiO_2$ and $LiNiO_2$-raw at the O *K* edges. The latter shows a clear spectral weight at the same energy of pre-edge peak of NiO.

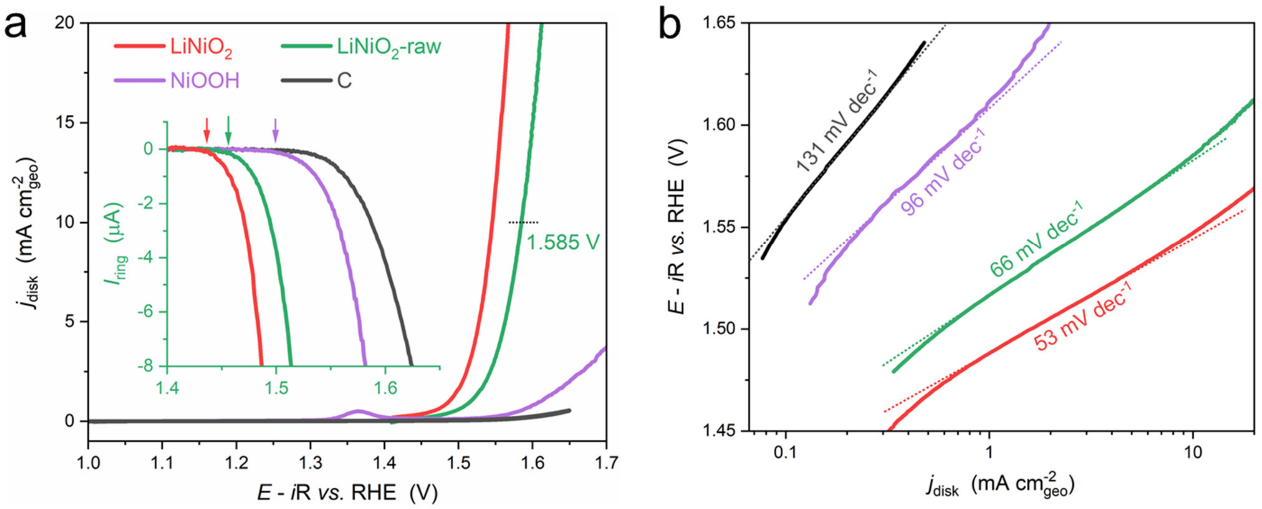

Supplementary Figure 18. (**a**–**b**) electrochemical measurements of $LiNiO_2$-0 (a $LiNiO_2$ sample without the second annealing step in high pressure O_2). (**a**) Linear sweep voltammograms and (**b**) Tafel plots of $LiNiO_2$-0, $LiNiO_2$, NiOOH, and carbon (Vulcan XC-72R) in Ar-saturated 1 M KOH solution, along with the corresponding Pt ring current shown in the inset (the onset potential of OER indicated by arrows). The voltammograms were collected under 1600 rpm with a sweep rate of 5 mV s^{-1}, and IR drops of the voltammograms were determined by EIS prior to the measurements, and compensated.

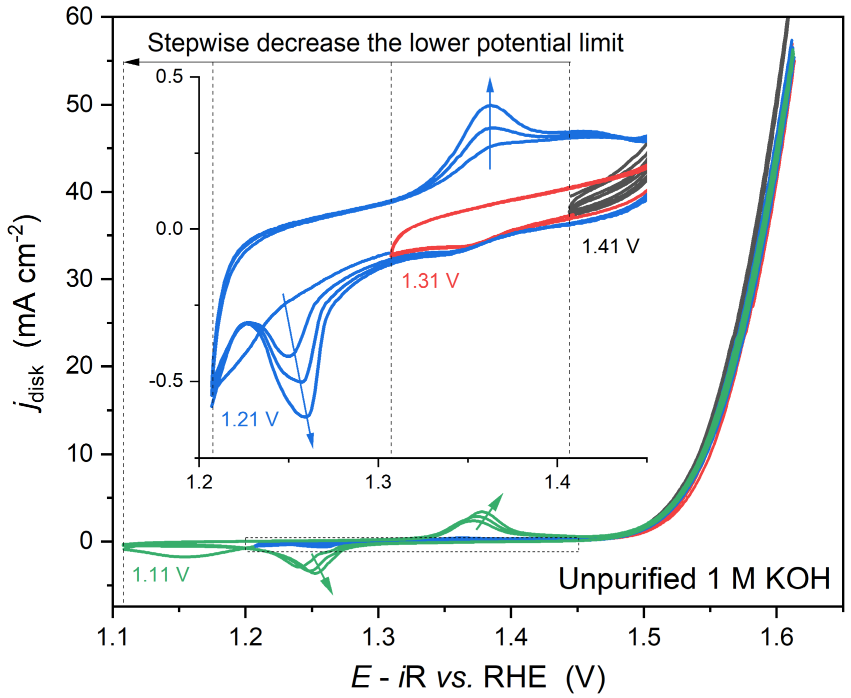

Supplementary Figure 19. Window-opening cyclic voltammograms of $LiNiO_2$ in unpurified 1 M KOH with stepwise decreasing the lower potential limits, showing the evolution of redox peaks (magnified in the corresponding insets). The voltammograms were collected under 1600 rpm with a sweep rate of 5 mV s^{-1}.

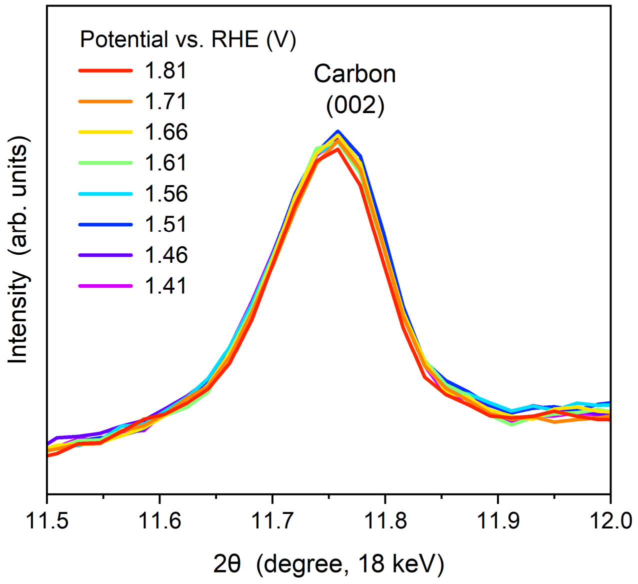

Supplementary Figure 20. *Operando* synchrotron XRD patterns of the as-prepared $LiNiO_2$ under applied potentials of 1.41–1.81 V at the carbon (002) peak region, showing the unchanged carbon signal during the operando XRD measurement.

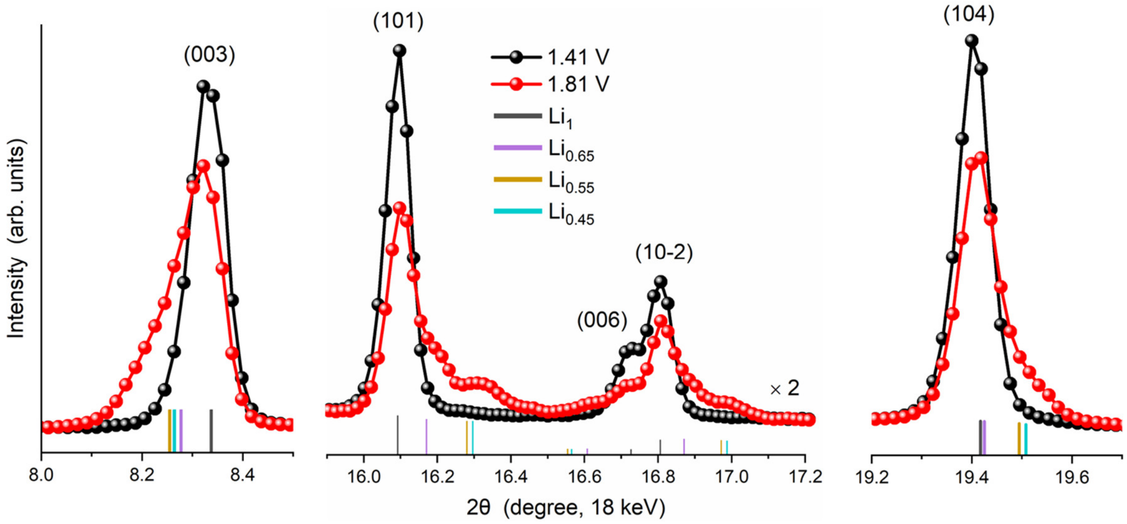

Supplementary Figure 21. Comparison of *operando* synchrotron XRD patterns of $LiNiO_2$ under 1.41 V and under 1.81 V. Standard patterns of $LiNiO_2$ (ICSD 78687) and delithiated Li_xNiO_2 (ICSD 78702 for x = 0.65, ICSD 78703 for x = 0.55 and ICSD 78704 for x = 0.45) are also shown for phase indexing.

Supplementary Table 5. ICP-MS results of Li concentration in 1 M KOH solution and Ni/Li molar ratio of the samples, before (0 min) and after $LiNiO_2$ delithiation at 1.41 V and 1.81 V.

	Li concentration (ng/ml)	Ni/Li molar ratio
0 min	0	1.0
1.41 V, 10 min [a]	6	1.2
1.41 V, 20 min [a]	6	1.2
1.41 V, 30 min [a]	6	1.2
1.81 V, 10 min	8	1.4
1.81 V, 20 min	10	1.6
1.81 V, 30 min	11	2.0

[a] Same as Li concentrations measured under OCP.

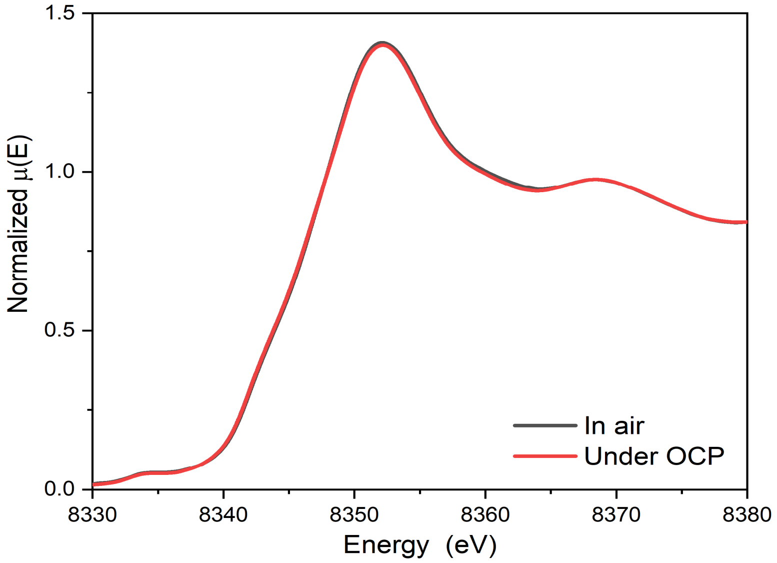

Supplementary Figure 22. Ni *K* edge XANES spectra of the *in situ* electrodes measured in air and under OCP, showing KOH electrolyte has no obvious effect on the XANES spectrum of $LiNiO_2$.

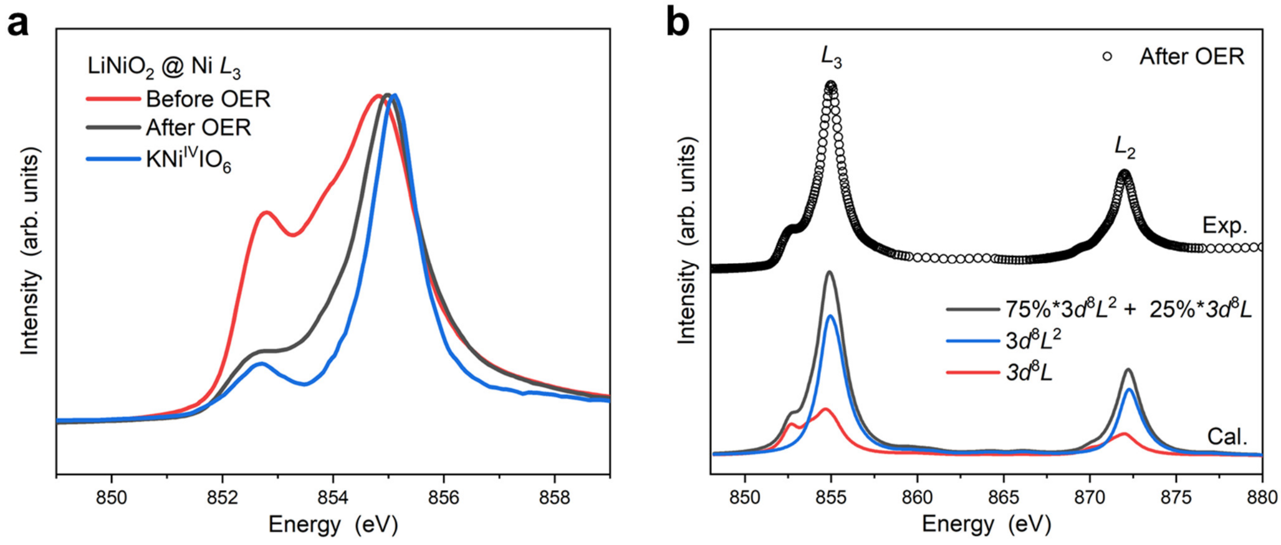

Supplementary Figure 23. **(a)** Ni $L_{3,2}$ sXAS of $LiNiO_2$ before and after OER and $KNiIO_6$ reference, and **(b)** total-electron-yield Ni $L_{3,2}$ sXAS of $LiNiO_2$ after OER (black circles), along with the sum (black line) of calculated spectra of 75% $3d^8L^2$ (blue line) and 25% $3d^8L$ (red line) configurations using the full multiplet cluster calculation[1].

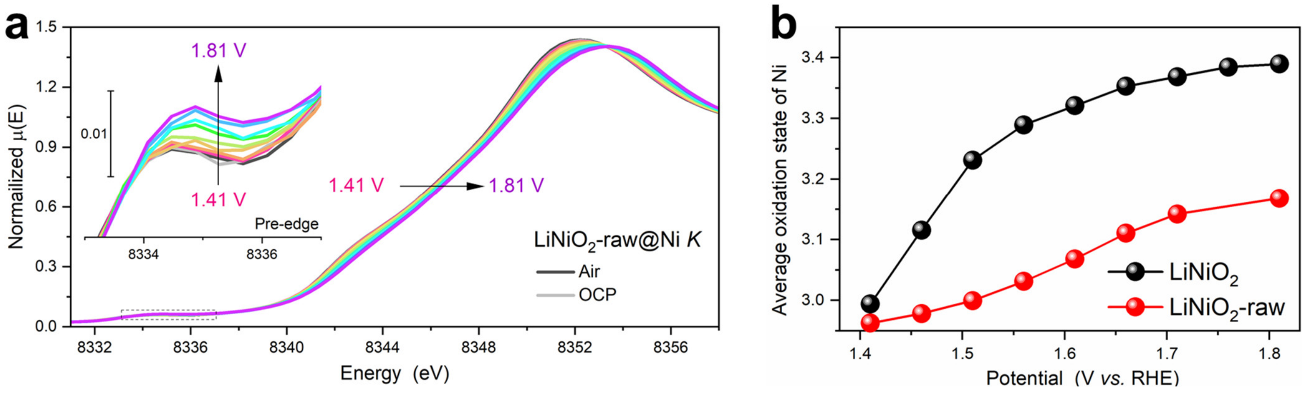

Supplementary Figure 24. **(a)** *Operando* XANES of $LiNiO_2$-raw at the Ni *K* edge, showing the potential dependent changes of the edge position and pre-edge, and **(b)** comparison of $LiNiO_2$ and $LiNiO_2$-raw in average oxidation states of Ni. The impeded Ni oxidation in $LiNiO_2$-raw is attribute to the structural transformation to $Ni^{III}OOH$ as observed in **Supplementary Figure 25.**

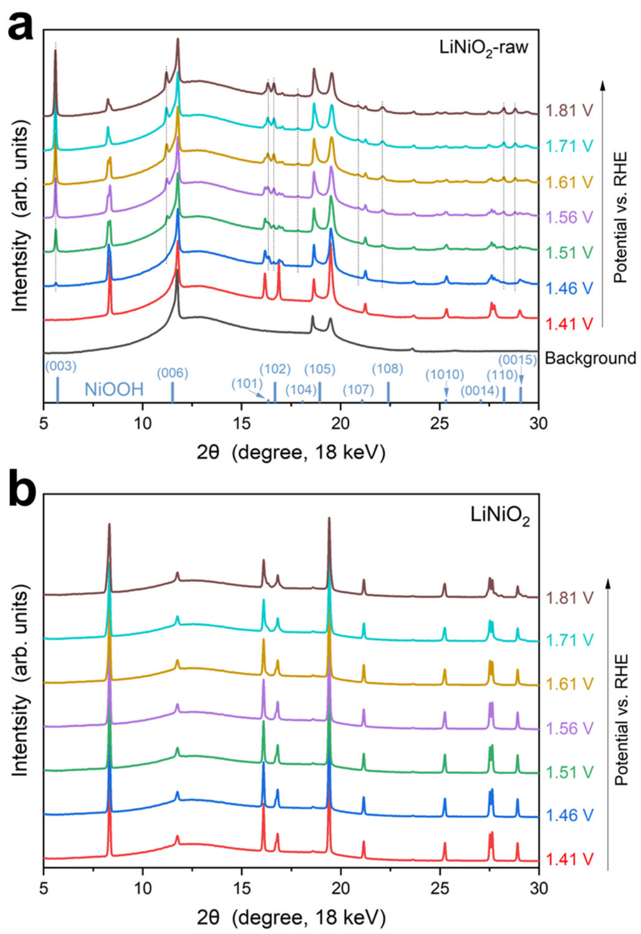

Supplementary Figure 25. *Operando* XRD patterns of **(a)** $LiNiO_2$-raw and **(b)** $LiNiO_2$ under applied potentials of 1.41–1.81 V in 1 M KOH electrolyte, showing increasing fraction of NiOOH phase with applied potentials. The background pattern without $LiNiO_2$-raw shows diffraction peaks of graphitic carbon and broad background from the electrolyte.

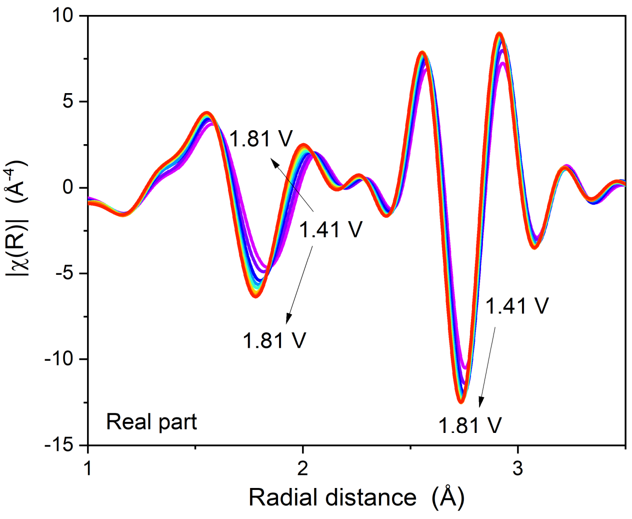

Supplementary Figure 26. Same as **Figure 6a**, but plotted in the real part of the Fourier transforms.

Supplementary Table 6. Structural parameters of $LiNiO_2$ and Ni references, obtained from EXAFS fitting. Plots and the corresponding fits of 1.41 V, 1.61 V and 1.81 V data are shown in **Supplementary Figure 27**, and those of constrained fits of 1.81 V data are shown in **Supplementary Figure 28**.

Sample	Scattering path[a]	*R* (Å)	N	σ^2 (x10^{-3} Å^2)	R factor (%)
**1.41 V**	Ni – O	1.955(5)	5.8(6)	12(2)	0.25
	Ni – Ni	2.877(4)	4.3(4)	4.9(5)	
**1.46 V**	Ni – O	1.943(4)	5.8(6)	12(1)	0.21
	Ni – Ni	2.876(3)	4.9(3)	5.3(4)	
**1.51 V**	Ni – O	1.933(4)	5.5(5)	11(1)	0.25
	Ni – Ni	2.874(4)	5.3(3)	5.4(4)	
**1.56 V**	Ni – O	1.924(4)	5.4(5)	10(1)	0.27
	Ni – Ni	2.869(4)	5.4(3)	5.4(3)	
**1.61 V**	Ni – O	1.920(4)	5.3(5)	10(1)	0.31
	Ni – Ni	2.866(4)	5.4(3)	5.4(5)	
**1.66 V**	Ni – O	1.914(4)	5.2(5)	9(1)	0.32
	Ni – Ni	2.862(4)	5.3(3)	5.3(5)	
**1.71 V**	Ni – O	1.912(4)	5.2(5)	9(1)	0.30
	Ni – Ni	2.861(4)	5.4(3)	5.3(4)	
**1.76 V**	Ni – O	1.910(4)	5.0(4)	8(1)	0.27
	Ni – Ni	2.860(4)	5.3(3)	5.2(4)	
**1.81 V**	Ni – O	1.909(4)	5.0(4)	8(1)	0.28
	Ni – Ni	2.860(4)	5.3(3)	5.3(4)	
**1.81 V, CN(Ni-O) = 6**	Ni – O	1.909(5)	6 [b]	10.7(6)	0.56
	Ni – Ni	2.857(4)	5.3(3)	5.3(4)	
**1.81 V, CN(Ni-O) = 5**	Ni – O	1.909(4)	5 [b]	8.2(4)	0.29
	Ni – Ni	2.860(3)	5.3(3)	5.3(4)	

[a] to minimise the fitting variables and to simplify the discussion of [$Ni^{III}O_6$] distortion, only one Ni–O scattering path was used in the first coordination shell, and thus the bond distortion in [$Ni^{III}O_6$] octahedra is mainly reflected by the parameter σ^2(Ni–O).

[b] not allowed to vary, to challenge the fitting model and to validate the presence of the under-saturated Ni

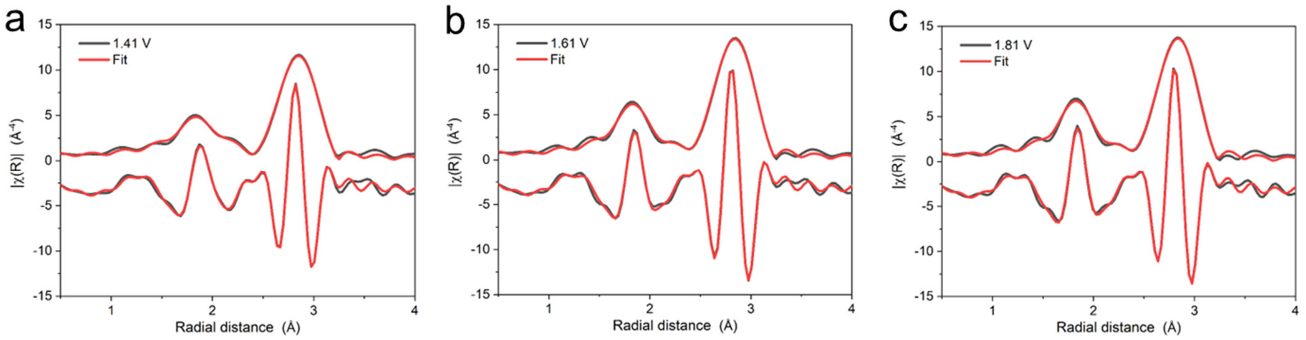

Supplementary Figure 27. Fits of *operando* EXAFS spectra of $LiNiO_2$ under applied potentials of (**a**) 1.41 V, (**b**) 1.61 V, and (**c**) 1.81 V. The fits are shown as the amplitude and the real part of *R* space. The Fourier transformation was k^3-weighted and performed over a *k* range of 3.5–13.6 $Å^{-1}$ with phase correction using the Ni–Ni scattering path and the fitting was carried out over a *R* range of 1–2.8 Å. The obtained structural parameters are listed in **Supplementary Table 6**, and the real part plots of the Fourier transform is shifted downwards for clarity.

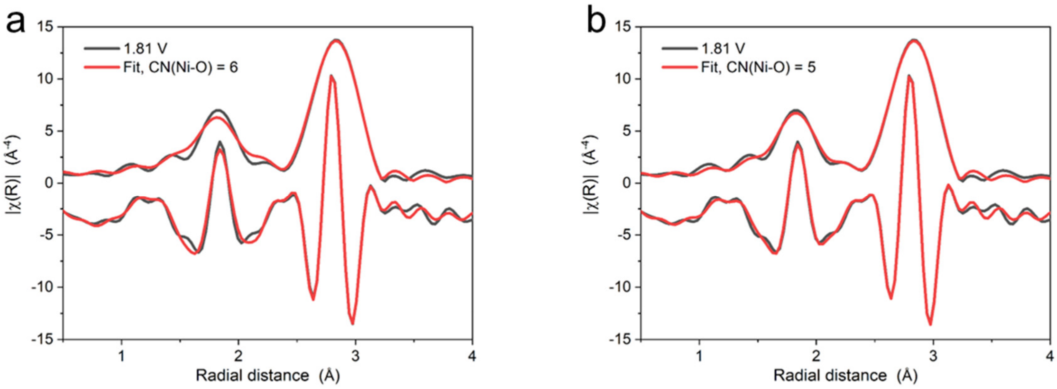

Supplementary Figure 28. Same as **Supplementary Figure 27**, but comparing EXAFS fits of the 1.81 V data with CN(Ni-O) being fixed (**a**) as 6 and (**b**) as 5. Forcing CN(Ni-O) = 6 ($LiNiO_2$ without oxygen vacancies) yields a worse fit than the fits where CN(Ni-O) is free and fixed to 5, in terms of R-factors (**Supplementary Table 6**) and reduced χ^2 (38 for the fit of CN = 6, 22 for the fit of CN = 5, and 28 when CN is a variable). The fit using CN(Ni-O) = 6 also produces an abnormal σ^2(Ni-O) of ~0.011 $Å^2$, which contradicts the oxidation of Ni^{III} to Ni^{IV} at high potentials.

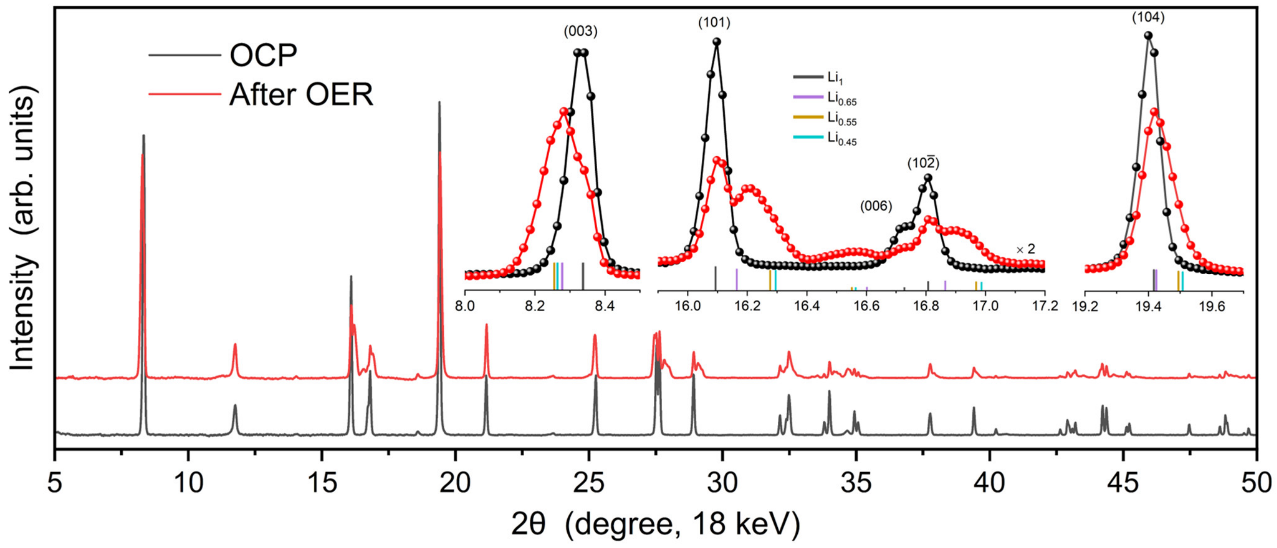

Supplementary Figure 29. XRD patterns of $LiNiO_2$ before and after OER measurements.

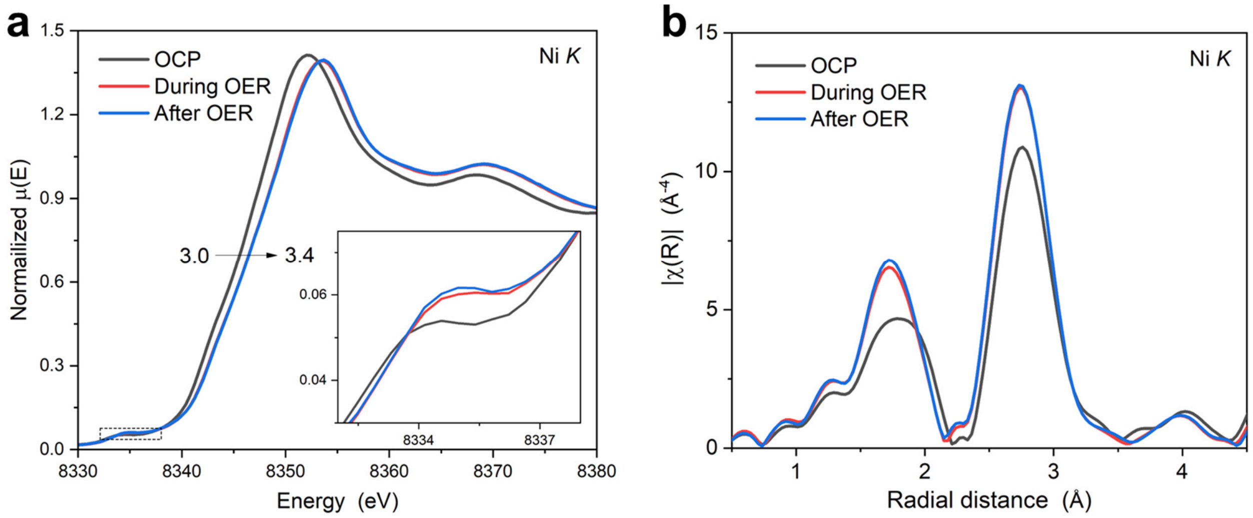

Supplementary Figure 30. **(a)** XANES and **(b)** EXAFS spectra of $LiNiO_2$ before, during and after OER measurements.

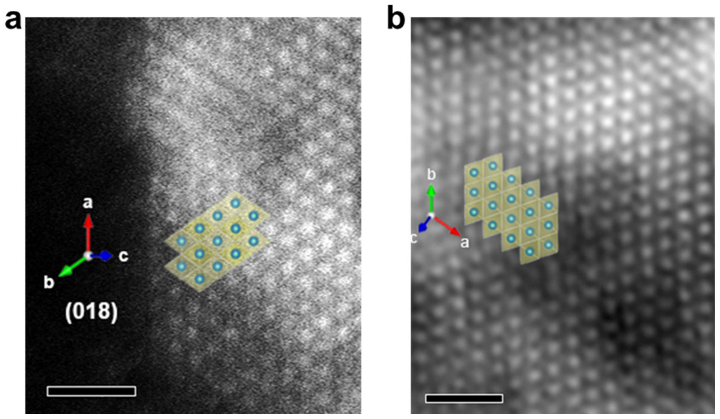

Supplementary Figure 31. Post-mortem AC-STEM images of $LiNiO_2$ after OER. These images compare the atomic arrangement of $LiNiO_2$ (a) at the surface and (b) in the bulk. Both show images perpendicular to the (018) lattice plane. The scale bar in b is 1 nm.

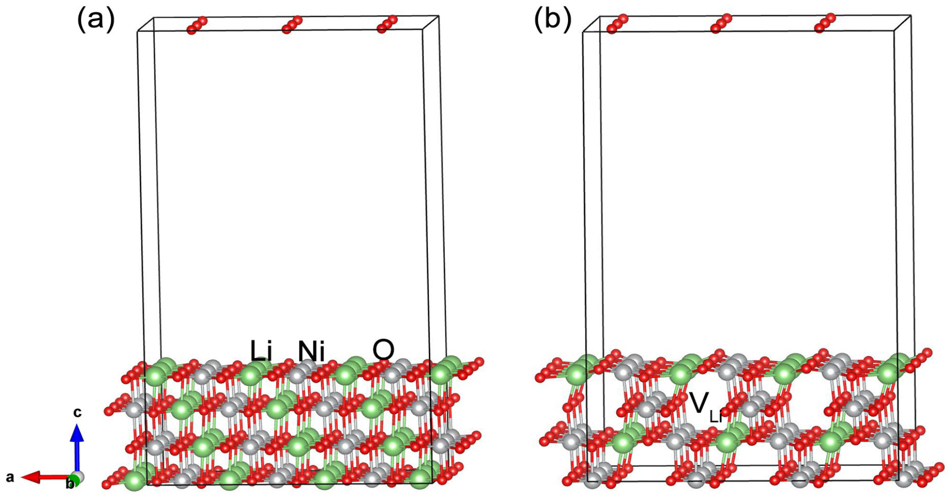

Supplementary Figure 32. The optimized local structures for the (102) surface of (a) $LiNiO_2$, and (b) $Li_{0.5}NiO_2$. The green, gray, and red balls indicate Li, Ni, and O respectively.

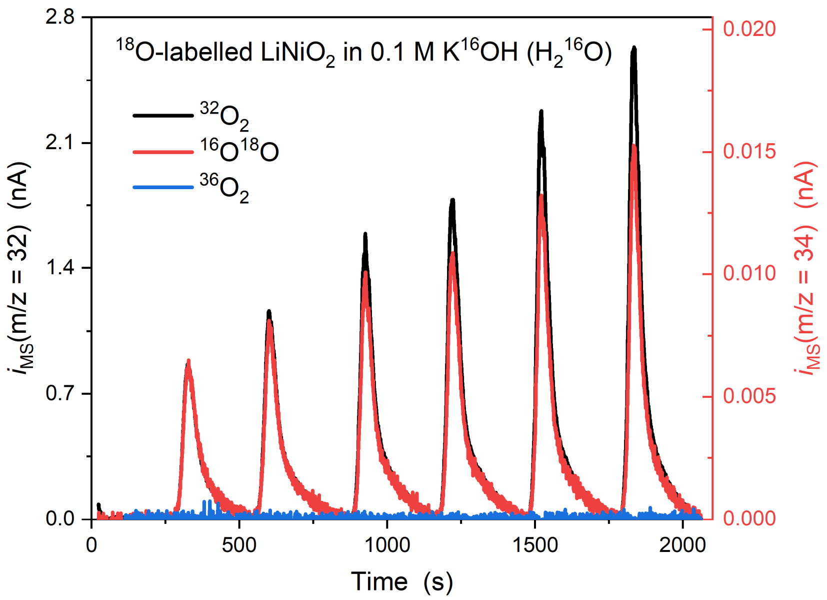

Supplementary Figure 33. MSCV curves of $^{32}O_2$, $^{16}O^{18}O$ and $^{36}O_2$ mass ion current of ^{18}O-labelled $LiNiO_2$ in ^{16}O-based 0.1 M KOH, plotted in the time domain, showing that the ratio between i_{MS}(m/z = 32) and i_{MS}(m/z = 34) increase with CV cycles. The CV cycles were carried out from 1.4 V to 1.8 V with a scan rate of 5 mV s^{-1}.

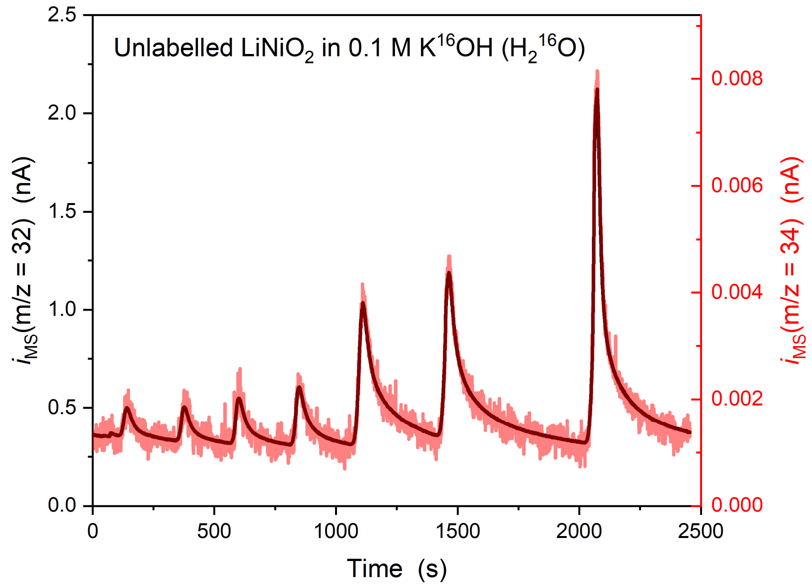

Supplementary Figure 34. MSCV curves of $^{32}O_2$, $^{16}O^{18}O$ and $^{36}O_2$ mass ion current of unlabelled $LiNiO_2$ in ^{16}O-based 0.1 M KOH, plotted in the time domain, showing that the ratio between i_{MS}(m/z = 32) and i_{MS}(m/z = 34) remain constant with CV cycles.

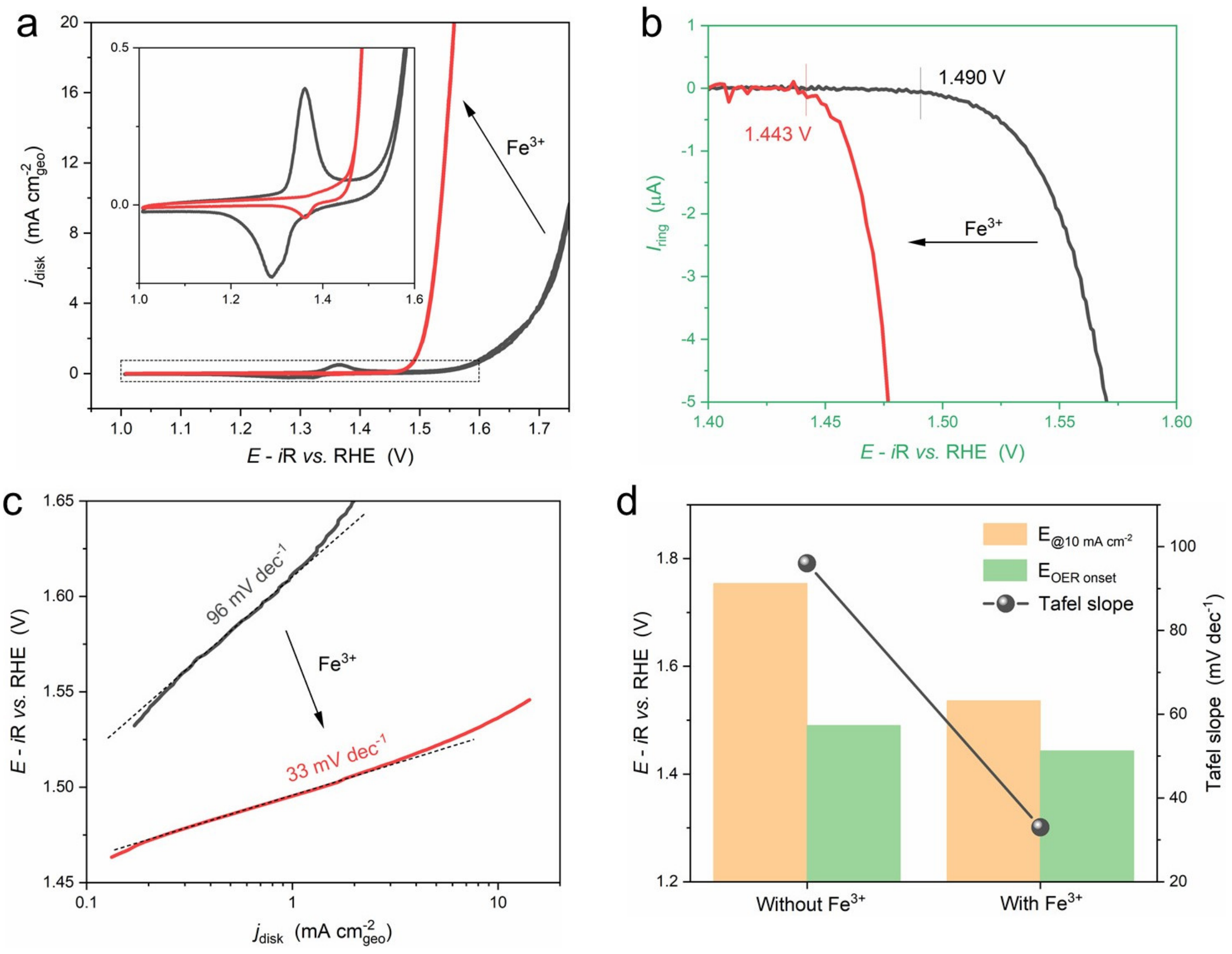

Supplementary Figure 35. Effects of Fe^{3+} on the OER activity of NiOOH. (**a**) CVs, (**b**) ring current and (**c**) Tafel plots with and without 100 μM Fe^{3+}. (**d**) A bar plot showing effects of Fe^{3+}on potential achieving 10 mA cm^{-2}, OER onset potentials and Tafel slopes.

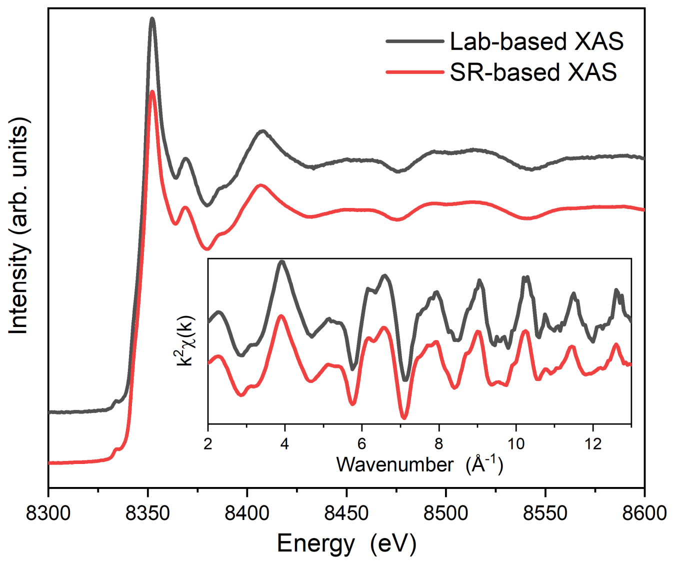

Supplementary Figure 36. Normalized Ni *K* edge XAS of $LiNiO_2$ measured using an in-house lab-based X-ray absorption spectrometer.

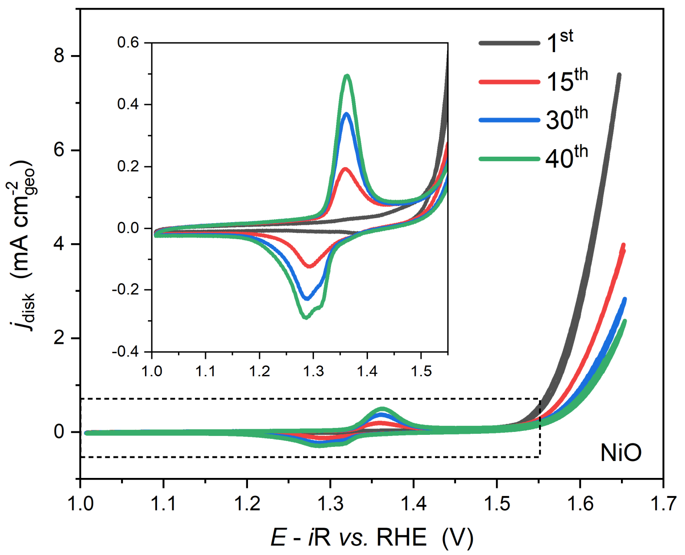

Supplementary Figure 37. Cyclic voltammograms of NiO in 1 M KOH with a scan rate of 5 mV s^{-1}. The surface redox peaks of Ni^{2+}/Ni^{III}, characteristics of $Ni(OH)_2$/NiOOH, develops with the scan number, indicating the transformation of NiO to NiOOH during CV in 1 M KOH.

Supplementary References

1. Parameters used in the cluster calculations (eV): U_{dd} = 6.7, U_{cd} = 8.7, $pd\sigma = -1.5$, Δ = -0.5 for Ni^{III} and Δ = -3.5 eV for Ni^{IV}; Slater integrals were set as 70% of Hartree-Fock values.

2. Ren, Y., et al. The effect of cation mixing in $LiNiO_2$ toward the oxygen evolution reaction. *ChemElectroChem* **8**, 70-76 (2020).

3. Li, N., et al. Unraveling the cationic and anionic redox reactions in a conventional layered oxide cathode. *ACS Energy Lett.* **4**, 2836-2842 (2019).

4. Fu, G., et al. Tuning the electronic structure of NiO via Li doping for the fast oxygen evolution reaction. *Chem. Mater.* **31**, 419-428 (2018).

5. Uchimoto, Y., Sawada, H. & Yao, T. Changes in electronic structure by Li ion deintercalation in $LiNiO_2$ from nickel L-edge and O K-edge XANES. *J. Power Sources* **97-98**, 326-327 (2001).

6. Zhu, K., et al. Layered Fe-substituted $LiNiO_2$ electrocatalysts for high-efficiency oxygen evolution reaction. *ACS Energy Lett.* **2**, 1654-1660 (2017).

7. Ren, X., et al. Constructing an adaptive heterojunction as a highly active catalyst for the oxygen evolution reaction. *Adv. Mater.* **32**, e2001292 (2020).

8. Wu, J., et al. Electrochemical water splitting by pseudo-spinel, disordered and layered lithium nickel oxides: correlation between structural motifs and catalytic properties. *ChemCatChem* **10**, 2551-2557 (2018).

9. Klaus, S., Cai, Y., Louie, M. W., Trotochaud, L. & Bell, A. T. Effects of Fe electrolyte impurities on $Ni(OH)_2$/NiOOH structure and oxygen evolution activity. *J. Phys. Chem. C* **119**, 7243-7254 (2015).

10. Mefford, J. T., et al. Correlative operando microscopy of oxygen evolution electrocatalysts. *Nature* **593**, 67-73 (2021).

11. Trotochaud, L., Young, S. L., Ranney, J. K. & Boettcher, S. W. Nickel-iron oxyhydroxide oxygen-evolution electrocatalysts: the role of intentional and incidental iron incorporation. *J. Am. Chem. Soc.* **136**, 6744-6753 (2014).

12. Burke, M. S., et al. Revised Oxygen Evolution Reaction Activity Trends for First-Row Transition-Metal (Oxy)hydroxides in Alkaline Media. *J. Phys. Chem. Lett.* **6**, 3737-3742 (2015).

13. Li, H., et al. Active Phase on $SrCo_{1-x}Fe_xO_{3-\delta}$ ($0 \leq x \leq 0.5$) Perovskite for Water Oxidation: Reconstructed Surface versus Remaining Bulk. *JACS Au* **1**, 108-115 (2021).

14. Lopes, P. P., et al. Dynamically Stable Active Sites from Surface Evolution of Perovskite Materials during the Oxygen Evolution Reaction. *J. Am. Chem. Soc.* **143**, 2741-2750 (2021).

15. Zhu, Y., et al. A High-Performance Electrocatalyst for Oxygen Evolution Reaction: $LiCo_{0.8}Fe_{0.2}O_2$. *Adv. Mater.* **27**, 7150-7155 (2015).

16. Zhou, J., et al. Voltage- and time-dependent valence state transition in cobalt oxide catalysts during the oxygen evolution reaction. *Nat. Commun.* **11**, 1984 (2020).

17. Maiyalagan, T., Jarvis, K. A., Therese, S., Ferreira, P. J. & Manthiram, A. Spinel-type lithium cobalt oxide as a bifunctional electrocatalyst for the oxygen evolution and oxygen reduction reactions. *Nat. Commun.* **5**, 3949 (2014).

18. Augustyn, V., Therese, S., Turner, T. C. & Manthiram, A. Nickel-rich layered $LiNi_{1-x}M_xO_2$(M = Mn, Fe, and Co) electrocatalysts with high oxygen evolution reaction activity. *J. Mater. Chem. A* **3**, 16604-16612 (2015).

19. Bak, J., Bin Bae, H. & Chung, S. Y. Atomic-scale perturbation of oxygen octahedra via surface ion exchange in perovskite nickelates boosts water oxidation. *Nat. Commun.* **10**, 2713 (2019).

20. Lu, Z., et al. Electrochemical tuning of layered lithium transition metal oxides for improvement of oxygen evolution reaction. *Nat. Commun.* **5**, 4345 (2014).

21. Yamada, I., et al. Bifunctional Oxygen Reaction Catalysis of Quadruple Manganese Perovskites. *Adv. Mater.* **29**, 1603004 (2017).

22. Huang, Z. F., et al. Tuning of lattice oxygen reactivity and scaling relation to construct better oxygen evolution electrocatalyst. *Nat. Commun.* **12**, 3992 (2021).
